# Ergebnisqualität und Kosten der allgemeinen und spezialisierten Palliativversorgung in Deutschland im regionalen Vergleich: eine GKV-Routinedatenstudie

**DOI:** 10.1007/s00103-023-03746-9

**Published:** 2023-08-03

**Authors:** Antje Freytag, Franziska Meissner, Markus Krause, Thomas Lehmann, Maximiliane Katharina Jansky, Ursula Marschall, Andreas Schmid, Nils Schneider, Horst Christian Vollmar, Ulrich Wedding, Bianka Ditscheid

**Affiliations:** 1 Institut für Allgemeinmedizin, Universitätsklinikum Jena, Bachstr. 18, 07743 Jena, Deutschland; 2 Zentrum für Klinische Studien, Universitätsklinikum Jena, Jena, Deutschland; 3 Klinik für Palliativmedizin, Universitätsmedizin Göttingen, Göttingen, Deutschland; 4 BARMER Institut für Gesundheitssystemforschung, Wuppertal, Wuppertal, Deutschland; 5 Gesundheitsökonomie und -management, Rechts- und Wirtschaftswissenschaftliche Fakultät, Universität Bayreuth, Bayreuth, Deutschland; 6 Oberender AG, Bayreuth, Bayreuth, Deutschland; 7 Institut für Allgemeinmedizin und Palliativmedizin, Medizinische Hochschule Hannover, Hannover, Deutschland; 8 Abteilung für Allgemeinmedizin (AM RUB), Medizinische Fakultät, Ruhr-Universität Bochum, Bochum, Deutschland; 9 Abteilung Palliativmedizin der Klinik für Innere Medizin II, Universitätsklinikum Jena, Jena, Deutschland

**Keywords:** Versorgung am Lebensende, Versorgungsqualität, Kosten-Effektivität, Sekundärddaten, Kleinräumige Analysen, End-of-life care, Quality of care, Cost-effectiveness, Secondary data, Small-scale analysis

## Abstract

**Hintergrund:**

Wesentliche Rahmenbedingungen für Palliativversorgung (PV) werden auf regionaler Ebene gesetzt. Der Umfang zum Einsatz kommender Versorgungsformen (ambulant, stationär, allgemein, spezialisiert) variiert regional stark. Welche Ergebnisqualität zu welchen Kosten wird mit der in einer KV-Region (Kassenärztliche Vereinigung) angebotenen PV erreicht?

**Methoden:**

Retrospektive Beobachtungsstudie mit BARMER-Routinedaten von 145.372 im Zeitraum 2016–2019 Verstorbenen mit PV im letzten Lebensjahr. Vergleich der KV-Regionen hinsichtlich folgender Outcomes: Anteil palliativ versorgter Menschen, die im Krankenhaus verstarben, potenziell belastende Versorgung in den letzten 30 Lebenstagen (Rettungsdiensteinsätze, [intensivmedizinische] Krankenhausaufenthalte, Chemotherapien, Anlage/Wechsel einer PEG-Sonde, parenterale Ernährung), Gesamtversorgungskosten der letzten 3 Lebensmonate, Kosten der PV(‑Formen) des letzten Lebensjahres, Kosten-Effektivitäts-Relationen sowie Patienten‑/Wohnkreismerkmals-adjustierte Kennzahlen.

**Ergebnisse:**

Die KV-Regionen variierten hinsichtlich der Outcomes (auch adjustiert) der PV deutlich. Über alle Outcomes aggregiert wies Westfalen-Lippe bessere Ergebnisse auf. Die PV-Kosten variierten ebenfalls stark, am stärksten bei spezialisierter ambulanter PV (SAPV). Die günstigste Kosten-Effektivitäts-Relation von Gesamtversorgungskosten zur Sterberate in der Häuslichkeit wies Westfalen-Lippe auf.

**Fazit:**

Regionen mit besserer Ergebnisqualität und günstigerer Kosten-Effektivität können Orientierung für andere Regionen bieten. Es sollte überprüft werden, inwieweit der neue SAPV-Bundesrahmenvertrag die empirischen Erkenntnisse aufgreifen kann. Patientenrelevanten Outcomes sollte stärkeres Gewicht gegeben werden als Parametern, die auf Versorgungsstrukturen abzielen.

**Zusatzmaterial online:**

Zusätzliche Informationen sind in der Online-Version dieses Artikels (10.1007/s00103-023-03746-9) enthalten.

## Abstract (en)

**Background:**

The main framework conditions for palliative care are set at the regional level. The scope of the forms of care used (outpatient, inpatient, general, specialized) varies widely. What is the quality of outcomes achieved by the palliative care provided on a federal states level? What are the associated costs of care?

**Method:**

Retrospective observational study using BARMER claims data from 145,372 individuals who died between 2016 and 2019 and had palliative care in the last year of life. Regional comparison with regard to the following outcomes: proportion of palliative care patients who died in the hospital, potentially burdensome care in the last 30 days of life (ambulance calls, [intensive care] hospitalizations, chemotherapy, feeding tubes, parenteral nutrition), total cost of care (last three months), cost of palliative care (last year), and cost-effectiveness ratios. Calculation of patient/resident characteristic adjusted rates, costs, and ratios.

**Results:**

Federal states vary significantly with respect to the outcomes (also adjusted) of palliative care. Palliative care costs vary widely, most strongly for specialized outpatient palliative care (SAPV). Across all indicators and the cost-effectiveness ratio of total cost of care to at-home deaths, Westphalia-Lippe shows favorable results.

**Conclusion:**

Regions with better quality and more favorable cost (ratios) can provide guidance for other regions. The extent to which the new federal SAPV agreement can incorporate the empirical findings should be reviewed. Patient-relevant outcome parameters should be given greater weight than parameters aiming at structures of care.

## Einführung

Hospizliche und palliative Versorgung findet in Deutschland ambulant oder stationär, allgemein oder spezialisiert statt. Entsprechend ihrer Identifizierungsmöglichkeit in (Routine‑)Abrechnungsdaten der Gesetzlichen Krankenversicherung (GKV) anhand verfügbarer Abrechnungsziffern wird zwischen den folgenden 5 Hauptversorgungsformen unterschieden:

- *AAPV* (allgemeine ambulante Palliativversorgung) wird vor allem von Hausärzten geleistet. Dafür sind entsprechende Gebührenordnungspositionen (GOP) in den Einheitlichen Bewertungsmaßstab (EBM) aufgenommen worden, die für Haus- und Kinderärzte abrechnungsfähig sind. Andere Facharztgruppen können AAPV-Leistungen nach Onkologie-Vereinbarung [1] oder über Selektivverträge (wenn vorhanden) abrechnen. Besonderheiten gelten z. B. in Nordrhein, wo Qualifizierte Palliativärzte (QPA) AAPV-Leistungen abrechnen können, sowie in Westfalen-Lippe, wo am dortigen Palliativversorgungsmodell [2] teilnehmende (Haus‑)Ärzte gesonderte AAPV-Ziffern abrechnen können.
- *BQKPmV* (besonders qualifizierte und koordinierte palliativmedizinische Versorgung) wurde im Jahr 2017 als weitere ambulante Form der Palliativversorgung eingeführt. Leistungen der BQKPmV können – nach Beantragung und Qualifikationsnachweis bei der zuständigen Kassenärztlichen Vereinigung (KV) – arztgruppenübergreifend abgerechnet werden [3].
- *SAPV* (spezialisierte ambulante Palliativversorgung) ist für Personen mit komplexem Symptomgeschehen und besonderem palliativmedizinischen Versorgungsbedarf eingeführt worden und wird durch SAPV-Teams erbracht [4]; die von den ambulanten palliativmedizinischen Konsiliardiensten (PKD) in Westfalen-Lippe erbrachten Palliativleistungen [2] werden in dieser Studie ebenfalls als spezialisierte ambulante Palliativleistungen betrachtet.
- *Stationäre Palliativversorgung*, sowohl geleistet auf Palliativstationen als auch durch den palliativmedizinischen Dienst in Krankenhäusern.
- *Stationäre Hospizleistungen*.^1^

Darüber hinaus können auch Leistungen der häuslichen Krankenpflege (HKP) gemäß Sozialgesetzbuch V sowie Pflegeleistungen gemäß Sozialgesetzbuch XI palliativer Art sein.

Detailliert beschrieben finden sich die genannten Versorgungsformen und entsprechende Aufgriffskriterien in [5] im dortigen Onlinematerial, Abschnitt A.3.

Für die Weiterentwicklung der Strukturen hospizlicher und palliativer Versorgung (PV) in Deutschland ist die Ebene der Bundesländer bzw. der kassenärztlichen Vereinigungen (KV) von besonderer Bedeutung, da wesentliche Rahmenbedingungen auf dieser Ebene festgelegt werden. So unterliegt beispielsweise die stationäre PV der föderalen Krankenhausplanung. Verträge zur SAPV gemäß § 37b und § 132d SGB V sind häufig KV-spezifisch [6, 7]. Für die Vergütung der AAPV gelten neben bundeseinheitlichen Vergütungsziffern teilweise KV-spezifische Vergütungen. Es ist deshalb naheliegend, von KV-spezifischen Hospiz- und Palliativversorgungsmodellen zu sprechen.

Innerhalb dieser KV-spezifischen Rahmenbedingungen ist die Inanspruchnahme insbesondere von spezialisierten PV-Formen in den vergangenen Jahren stetig angestiegen, variiert jedoch stark zwischen den KV-Regionen [5, 8]. Dabei zeigte sich auch, dass die zum Einsatz kommenden Formen der PV nur bedingt von medizinischer Notwendigkeit bestimmt sind, da die Variabilität zwischen den KV-Regionen kaum über versorgungsbedarfs- und versorgungszugangsbezogene Merkmale der Patienten, die dort ihren Wohnort haben, erklärbar ist [5]. Neben den Kriterien des (objektiven) Bedarfs dürfte daher die regionale Verfügbarkeit eine bestimmende Rolle für die Wahl der Versorgungsform spielen [9, 10].

Welche Ergebnisse aber werden mit den in ihren Rahmenbedingungen und Inanspruchnahmestrukturen regional so unterschiedlichen PV-Modellen erreicht, mit Blick auf patientenrelevante Ergebnisqualität und dafür eingesetzte Kosten? Derartige empirische Informationen werden zur zielorientierten Weiterentwicklung der Hospiz- und Palliativversorgungsstrukturen in Deutschland dringend benötigt. Eine erste deutschlandweite Bestandsaufnahme dazu stammt aus dem Jahr 2015 [11]. Die vorliegende Studie liefert einen umfassenderen, belastbareren und aktualisierten Überblick über die Qualität der PV in den 17 KV-Regionen Deutschlands und untersuchte darüber hinaus auch die damit verbundenen Kosten. Eigene Vorarbeiten dazu stammen aus dem Jahr 2020 [12].

Für die Messung patientenrelevanter Ergebnisqualität von PV wird in Anlehnung an die Literatur [13, 14] davon ausgegangen, dass ein Versterben in der häuslichen Umgebung dem Versterben im Krankenhaus in der Regel vorgezogen wird und mithin ein höherer Anteil von in der Häuslichkeit Verstorbenen eine höhere Qualität der PV impliziert. Zudem soll eine auf die Erhöhung der Lebensqualität am Lebensende gerichtete PV empfehlungsgemäß mit einem Verzicht auf vermeidbare besonders aggressive, intensive bzw. belastende Therapien einhergehen [11, 15–23]. Inwieweit dies gelang, wurde anhand einer Reihe von einschlägigen Indikatoren gemessen.

Zusätzlich kann die Vermeidung aggressiver Therapien am Lebensende, weil diese häufig auch kostenintensiv sind (z. B. Krankenhausaufenthalte), trotz der für die PV aufgewendeten Kosten insgesamt zu Gesamtkosteneinsparungen führen. Solche Kosteneinsparungen wurden in internationalen Studien bereits vielfach gezeigt [16, 17, 24, 25], andere Studien fanden diese nicht [26, 27]. Die vorliegende Studie ist jedoch nicht auf den Nachweis von Kosteneinsparungen durch PV, sondern auf den regionalen Vergleich der Gesamtversorgungskosten, inkl. der Kosten der PV, gerichtet.

## Methoden

In einer retrospektiven Beobachtungsstudie mit Routinedaten der BARMER wurden Versicherte (VS) mit Sterbedatum in den Jahren 2016–2019 betrachtet, bei denen in ihrem letzten Lebensjahr mindestens eine ambulante, stationäre (auch hospizliche), allgemeine und/oder spezialisierte palliative Versorgungsleistung abgerechnet wurde (siehe Onlinematerial, Abschnitt A, [5]).

Nur VS mit PV wurden in die Analysen eingeschlossen. VS, die im letzten Lebensjahr keine PV erhielten, wurden nicht eingeschlossen, weil anhand der uns vorliegenden Daten keine valide Aussage darüber getroffen werden kann, ob für diese VS überhaupt PV-Bedarf bestanden hat bzw. sie von PV profitiert hätten. Aus diesem Grund haben wir auf eine Ausweisung der Outcomes für VS ohne PV verzichtet. Im Sinne einer umfassenden Gesamtbetrachtung werden allerdings die KV-spezifischen Raten der VS ohne PV zur Beurteilung eines möglichen Selektionseffekts berichtet. Ein solcher läge vor, wenn gute (schlechte) Outcomes bei VS mit PV in einer Region darauf zurückzuführen wären, dass viele (wenige) VS nicht palliativ versorgt wurden.

Zur Beurteilung der (Ergebnis‑)Qualität wurden die KV-Regionen hinsichtlich des Sterbeortes der dort wohnhaften VS mit PV sowie hinsichtlich potenziell vermeidbarer aggressiver Behandlung in den letzten 30 Lebenstagen verglichen. Im Einzelnen wurden 9 Indikatoren betrachtet, die in nationalen und internationalen Studien für die Beschreibung der (Ergebnis‑)Qualität bzw. Outcomes palliativer Versorgung am Lebensende herangezogen wurden und anhand von GKV-Routinedaten operationalisiert werden können [11, 15–21]: a) Versterben im Krankenhaus (insgesamt); b) Versterben im Krankenhaus, aber nicht auf Palliativstation; (folgende Punkte jeweils bezogen auf die letzten 30 Lebenstage:) c) Rettungsdiensteinsatz; d) Krankenhausaufenthalt; e) Krankenhausaufenthalt ohne palliativmedizinische Leistung; f) intensivmedizinischer Krankenhausaufenthalt; g) Chemotherapie; h) parenterale Ernährung und i) Anlage/Wechsel einer PEG-Sonde (Magensonde, perkutane endoskopische Gastrostomie). Ergänzend wurden diese Indikatoren je KV zu einem aggregierten Outcome als ungewichtetes arithmetisches Mittel der 9‑z-standardisierten Outcomes zusammengefasst.

Die Messung der ökonomischen Auswirkungen der PV erfolgte anhand der Gesamtversorgungskosten der letzten 3 Lebensmonate aus GKV-Perspektive. Berücksichtigte Leistungssektoren sind: ambulante ärztliche Versorgung, HKP (Häusliche Krankenpflege, SGB V), Krankenhaus, Rettungsdienst, Krankentransport, Hospiz, Arzneimittel, Heilmittel, Hilfsmittel, exkl. SGB-XI-Pflege. Darin enthalten sind auch die Palliativversorgungskosten der letzten 3 Lebensmonate. Die Rationale hinter der Zeitabgrenzung ist, dass PV im arithmetischen Mittel etwa 90 Tage vor dem Tod beginnt (Quelle: eigene Berechnungen, siehe Abb. B.1‑2 in Abschnitt B des Onlinematerials) und somit nicht etwa die Gesamtversorgungskosten im gesamten letzten Lebensjahr, sondern diejenigen Kosten der letzten 90 Lebenstage beeinflussen kann. Die Kosten der Palliativversorgung(sformen) des letzten Lebensjahres werden als Interventionskosten betrachtet.

Um Qualität und Kosten nicht nur nebeneinander, sondern auch im Verhältnis zueinander betrachten zu können, bildeten wir zusätzlich die Kosten-Effektivitäts-Relation (KER) auf der Ebene der KV-Regionen als Verhältnis zwischen Gesamtversorgungskosten und der Rate des Versterbens in der Häuslichkeit (entsprechend dem Nicht-Versterben im Krankenhaus). Im Ergebnis kann eine KV-Region also eine kosteneffektivere PV aufweisen, wenn sie mit hohen Versorgungskosten bei hoher Versterberate in der Häuslichkeit eine günstigere KER erreicht als eine KV-Region mit geringen Kosten, aber im Verhältnis geringerer Sterberate in der Häuslichkeit. Der gesundheitsökonomischen Methode der Kosten-Konsequenz-Analyse [28–30] folgend, verzichten wir auf die Ermittlung inkrementeller Kosten-Effektivitäts-Relationen. Es geht uns nicht um den inkrementellen Unterschied zwischen einer KV-Region und einer Referenz-KV-Region im Sinne einer „angemessenen Vergleichstherapie“, sondern um die deskriptive Zusammenschau der KER aller KV-Regionen.

Da die Vermutung naheliegt, dass regionale Unterschiede hinsichtlich der betrachteten Zielgrößen zum Teil über regionale Unterschiede in Morbidität und sozioökonomischen Faktoren erklärt werden können, wurden die Zielgrößen um relevante Einflussfaktoren adjustiert. Die verwendeten Adjustierungsfaktoren umfassen in GKV-Routinedaten verfügbare soziodemografische Merkmale und Patientencharakteristika aus dem letzten Lebensjahr sowie Merkmale des Wohnorts. Sie wurden bereits in einer vorangegangenen Studie zur Adjustierung der Inanspruchnahme von PV herangezogen [5] und hängen nicht nur mit der Inanspruchnahme palliativer Versorgung, sondern auch mit den qualitätsbezogenen Outcome-Indikatoren und Versorgungskosten am Lebensende signifikant zusammen (Tab. B.1-5). Die verwendeten Adjustierungsfaktoren sind:

- Alter (metrisch),
- Geschlecht (binär),
- Pflegebedarf (gemessen als dreistufige kategoriale Variable: Leben in der Häuslichkeit ohne Pflegegrad, Leben in der Häuslichkeit mit Pflegegrad bzw. Leben im Pflegeheim (Zeitpunkt: Beginn des letzten Lebensjahres; Pflegegrad: letzter dokumentierter Pflegegrad)),
- die aggregierte Morbidität (gemessen als gewichteter Charlson-Komorbiditätsindex, CCI),
- das Vorhandensein (dichotom) einer Krebs- und/oder einer anderen potenziell palliativversorgungsrelevanten chronischen Grunderkrankung (Dokumentation einer entsprechenden gesicherten ambulanten und stationären Haupt‑/Nebendiagnose im letzten Lebensjahr in Anlehnung an Murtagh et al. [31], Tabelle A.7-1) sowie
- als Merkmale des Versorgungszugangs: der sozioökonomische Deprivationsgrad (German Index of Socioeconomic Deprivation, GISD [32]) und der Anteil ländlich lebender Einwohner des Wohnkreises des Verstorbenen (beide metrisch).

Eine Limitation bei der Adjustierung ist sicherlich, dass wir andere denkbare Einflussfaktoren für qualitätsbezogene Outcomes und Kosten nicht erfassen konnten, auch wenn einige durchaus mit dem GISD abgedeckt sein dürften. Die Adjustierung erfolgte je nach Outcome- bzw. Kostenindikator mittels multipler logistischer bzw. linearer Regression.

Alle Analysen beruhen auf alters- und geschlechtsstandardisierten Daten, die auf einer Gewichtung anhand von je Bundesland und Jahr ermittelten Standardisierungsfaktoren basieren [5]. Details zur Methodik sind in Abschnitt A des Onlinematerials dargelegt.

## Ergebnisse

### Studienpopulation.

Von den 417.405 BARMER-VS mit Sterbedatum in 2016 bis 2019 wurden 145.372 (34,8%) VS im letzten Lebensjahr palliativ versorgt. Nur diese palliativ versorgten VS wurden in die Analysen zu Ergebnisqualität und Kosten eingeschlossen. Im Onlinematerial finden sich Übersichten zur Zahl der eingeschlossenen VS mit PV (und der nicht eingeschlossenen VS ohne PV) je KV-Region (Tab. B.1-1) sowie deren Zusammensetzung nach den Adjustierungsfaktoren (Tab. B.1‑2 bis B.1-4). Mit welchen Raten die eingeschlossenen VS je KV die unterschiedlichen Formen ambulanter, stationärer, allgemeiner und spezialisierter PV in Anspruch genommen haben, zeigen Abb. B.1‑1 und Tab. B.1‑6 im Onlinematerial.

### Qualitätsbezogene Outcomes.

**Figure 1:**
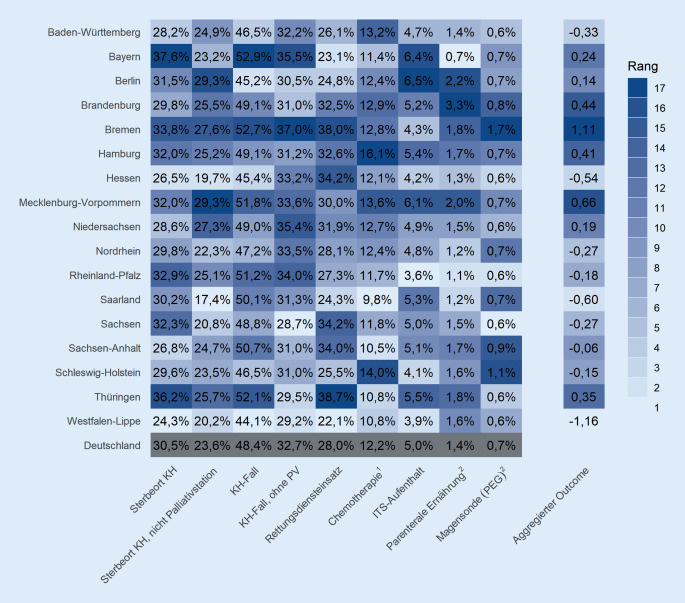

Abb. 1 enthält eine Ergebnisübersicht über alle qualitätsbezogenen Outcome-Indikatoren je KV-Region, wobei hier eine helle Einfärbung bessere Raten kodiert. Die Spannen zwischen dem jeweils ersten und letzten Rang sind deutlich. So variiert die Rate der im Krankenhaus verstorbenen VS von 24,3 % in Westfalen-Lippe bis 37,6 % in Bayern. Der Anteil an VS, die in den letzten 30 Lebenstagen einen Rettungsdiensteinsatz hatten, reicht von 22,1 % in Westfalen-Lippe bis 38,7 % in Thüringen. Auch andere Indikatoren zeigen eine erhebliche Variabilität. Werden die 9 Outcome-Indikatoren zu einer aggregierten Größe zusammengefasst, findet sich Westfalen-Lippe auf Rang 1, gefolgt vom Saarland und Hessen. Die letzten Ränge belegen Brandenburg, Mecklenburg-Vorpommern und Bremen.

**Figure 2:**
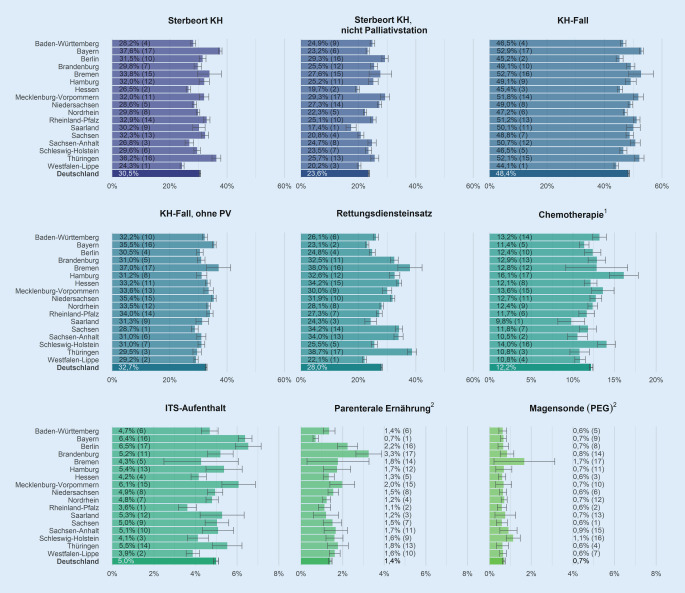

Die Konfidenzintervalle der qualitätsbezogenen Outcome-Indikatoren (Abb. 2) zeigen, dass die Datenbasis je KV-Region – mit Ausnahme von parenteraler Ernährung und dem Einsatz einer PEG-Sonde – ausreichend groß ist, um ernstzunehmende Aussagen über die zum Teil erhebliche regionale Variabilität in der Versorgungsqualität treffen zu können.

Dabei lässt sich die klinische Relevanz der Unterschiede in den qualitätsbezogenen Outcome-Indikatoren, die in allen VS mit PV gemessen wurden, anhand einer Hochrechnung auf die deutsche Gesamtbevölkerung verdeutlichen: Wenn 1 Prozentpunkt mehr Menschen in der Häuslichkeit versterben würden, wären das insgesamt jährlich ca. 3200 von etwa 320.000 Menschen in Deutschland, die im letzten Lebensjahr PV erhalten haben.

Nach Adjustierung der Outcome-Indikatoren zeigen sich nur wenige Veränderungen der Raten und Ränge (Abb. B.2-1/B.2-2). Wie aus Abb. B.2‑3 hervorgeht, weist Rheinland-Pfalz die deutlichste Verbesserung von Rang 7 auf 3 und Baden-Württemberg die deutlichste Verschlechterung von Rang 4 auf 11 auf. Unverändert bleiben Westfalen-Lippe auf Rang 1 und Bremen auf Rang 17.

### Gesamtversorgungskosten der letzten 3 Lebensmonate.

**Figure 3:**
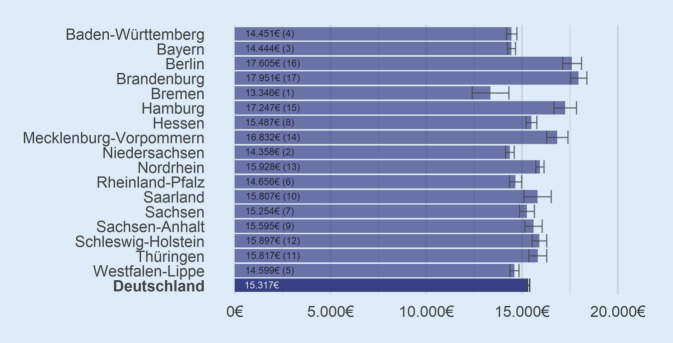

Die Gesamtversorgungskosten der letzten 3 Lebensmonate (Abb. 3) liegen im Bundesmittel je VS bei 15.317 € (SD: 14.757 €, Median: 11.654 €) zzgl. Pflegekosten gemäß SGB XI von 3522 € (SD: 2493 €, Median: 3312 €). Sie variieren zwischen den KV-Regionen deutlich von 13.346 € in Bremen bis 17.951 € in Brandenburg. Die Aufteilung der Gesamtkosten auf die berücksichtigten Leistungsbereiche nach KV-Regionen findet sich im Onlinematerial (Abb. B.3‑1, Tab. B.3-1/B.3-2). Nach Adjustierung der Gesamtkosten werden die Unterschiede zwischen den KV-Regionen insgesamt etwas reduziert, Bremen und Brandenburg bleiben auf dem ersten bzw. letzten Rang (Abb. B.3-2).

### Kosten der Palliativversorgung im letzten Lebensjahr.

**Figure 4:**
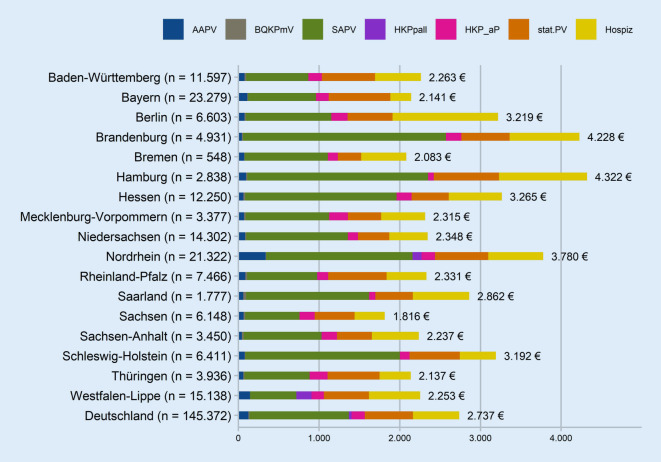

Die Zusammensetzung der Palliativversorgungskosten zeigt Abb. 4. Der größte Anteil entfällt auf die Kosten für SAPV. Zwischen den KV-Regionen variieren die Palliativversorgungskosten erheblich zwischen im Mittel 1816 € in Sachsen bis 4322 € in Hamburg (Tab. B.4-1/B.4-2). Auch nach Adjustierung der Palliativversorgungskosten belegen Sachsen und Bremen die ersten beiden Ränge, Hamburg und Brandenburg die letzten beiden (Abb. B.4-1).

Die bisherigen Ergebnisse hinsichtlich der Kosten pro Person basierten auf der gesamten Studienpopulation der VS mit PV im letzten Lebensjahr. Ergänzend dazu ermittelten wir die Kosten der palliativen Leistungen je KV-Region ausschließlich für jene VS, für die die jeweilige PV-Form tatsächlich abgerechnet wurde (Abb. B.4‑2 bis B.4-4). Dabei schwanken etwa die mittleren SAPV-Kosten je VS mit SAPV zwischen 739 € in Westfalen-Lippe und 5645 € in Nordrhein (Abb. B.4-2/B.4-5A), Median: zwischen 600 € in Westfalen-Lippe und 3300 € in Bayern (Abb. B.4-4). Wenn man die (außer in Berlin, Brandenburg, Mecklenburg-Vorpommern und Niedersachsen neben der SAPV verordenbare [6]) häusliche Krankenpflege hinzurechnet, reicht die Spanne von 1136 € in Westfalen-Lippe bis 5924 € in Nordrhein (Abb. B.4-2/B.4-5B). An diesem Bild ändert eine Adjustierung ebenfalls nichts (Abb. B.4-6A/B).

### Kosten-Effektivitäts-Relation.

**Figure 5:**
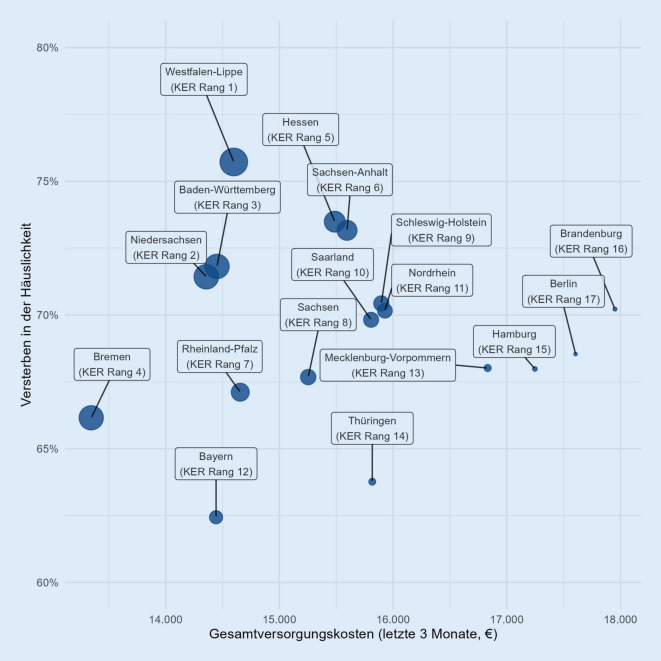

Bei der Relation zwischen den in den letzten 3 Lebensmonaten aufgewendeten Gesamtversorgungskosten und der Sterberate in der Häuslichkeit (KER) als Maß für die Höhe der am Lebensende aufgewendeten Versorgungskosten je erreichtem Prozent des Versterbens in der Häuslichkeit belegt Westfalen-Lippe den ersten Rang, Berlin den letzten (Abb. 5). Zieht man adjustierte Werte (Abb. B.5-1) heran, bleibt Westfalen-Lippe auf Rang 1, Mecklenburg-Vorpommern belegt nun den letzten Rang. Im Mittelfeld verschieben sich die Ränge einiger KV-Regionen.

### Berücksichtigung der nichtversorgten Versicherten.

Einen durch den Ausschluss von VS ohne PV aus den Analysen möglicherweise entstandenen Selektionsbias, weswegen gute (schlechte) Outcomes in KV-Regionen durch ein hohes (geringes) Maß an palliativer Unterversorgung begründet wären, stützen unsere Daten nicht: So bekleidet Sachsen-Anhalt beim höchsten Anteil an Nichtversorgten von 71,8 % einen mittleren Rang bei den Outcomes ebenso wie Bayern mit dem geringsten Anteil an Nichtversorgten von 55,3 % (Tab. B.1-1). Andererseits belegt das Outcome-stärkste Westfalen-Lippe einen mittleren Rang (Nr. 13) und das Outcome-schwächste Bremen den Rang 16 bei der Rate Nichtversorgter. Der genannte Selektionsbias ist entsprechend als geringfügig einzuschätzen.

## Diskussion

Die vorgelegte Studie liefert erstmals einen umfassenden Vergleich der in den 17 KV-Regionen Deutschlands geleisteten PV, die aus ambulanter und stationärer, allgemeiner und spezialisierter hospizlicher und palliativer Versorgung zusammengesetzt ist, hinsichtlich GKV-Routinedaten-basierter Outcomes zu patientenrelevanter Ergebnisqualität von PV sowie im Rahmen der GKV aufgewendeter Versorgungskosten.

Der Vergleich der herangezogenen Outcome-Indikatoren zur Abbildung der Qualität von PV [10, 11, 15, 22, 33] sowie der Wirkungen der PV auf die Versorgungskosten [34–36] mit den Ergebnissen anderer Studien kann dem Onlinematerial (Abschnitte C.1 und C.2) entnommen werden (externe Validität). Zu Palliativversorgungskosten sowie Kosten-Effektivitäts-Analysen liegen bislang (bis auf die KJ1-Statistik, [37]) nur ausländische Studien und Versorgungsform vergleichende Studien [38–40] vor.

### Limitationen und Stärken der Studie

Patienten-berichtete Outcomes (Schweregrad der Symptome, Patientenzufriedenheit, soziale Unterstützung), die bei individuellen Versorgungsentscheidungen von Bedeutung sind, sind in GKV-Routinedaten nicht verfügbar, was eine gravierende Limitation dieser Studie darstellt [15]. Trotz dieser (bekannten) Einschränkungen von GKV-Routinedaten weist die Studie gleichzeitig 2 erhebliche Stärken auf, die die Schwächen aufwiegen: Erstens sind die gewählten Zielgrößen sehr wohl als patientenorientiert zu bewerten – sie rechtfertigen es, von Ergebnisqualität zu sprechen. Zweitens ist die Studienpopulation mit etwa 150.000 Menschen von erheblicher Größe, in der auch selten vorkommende Ereignisse reliabel gemessen werden können – die Aussagekraft der Ergebnisse ist deshalb als hoch einzuschätzen.

Bei der Rangvergabe nach dem Prinzip „bester Rang für kleinste Rate“ kann der Eindruck entstehen, es existiere ein Optimum, das bei 0 % liege. Dem ist natürlich nicht so, da unstrittig bedarfsgerechte bzw. patientenorientierte Indikationen für in Anspruch genommene Behandlungen bestanden haben können. Der regionale Vergleich – und dies ist die besondere Stärke dieser Studie – hebt jedoch nicht auf absolute Werte, sondern auf die Unterschiede zwischen den KV-Regionen ab. Vergleichsweise geringe bzw. unterdurchschnittliche Raten können so tendenziell als Hinweis auf eine vergleichsweise bessere Versorgung gewertet werden. Nicht auszuschließen ist dabei, dass geringe Raten mit (zu) geringen Versorgungskapazitäten, sprich Unterversorgung, assoziiert sein könnten.

Weiterhin liegen gegenseitige Abhängigkeiten einiger Indikatoren vor, die Aggregation erfolgt ohne patientenorientierte Gewichtung der Indikatoren und relevante Kovariaten sind nicht vollständig erfasst. Für die Indikatorenausdifferenzierung, -aggregation/-gewichtung und -adjustierung sind somit Weiterentwicklungen angezeigt. Der hier eingeschlagene Weg legt dafür eine solide Basis.

Die Studienergebnisse sind trotz der vorgenommenen Alters- und Geschlechtsstandardisierung nur eingeschränkt auf die GKV-Population insgesamt bzw. in den jeweiligen KV-Regionen übertragbar. Dies ist zurückzuführen auf potenzielle Spezifika von BARMER-Versicherten hinsichtlich PV-relevanter Patientenmerkmale, die unterschiedlichen Anteile der BARMER-Versicherten an den Verstorbenen in den einzelnen KV-Regionen (die insbesondere in Bremen und im Saarland gering sind) sowie potenzielle Spezifika der durch die BARMER (bzw. Ersatzkassen) geschlossenen Selektivverträge zur PV.

Nachgeordnete Limitationen werden im Onlinematerial (C.3) beschrieben.

### Identifizierung von KV-Regionen mit Good Practice

Die Ergebnisse deuten mit einer Vielzahl gemessener Indikatoren und durchgeführter Analysen darauf hin, dass die KV-Region Westfalen-Lippe besonders vorteilhafte Ergebnisse erzielt, mithin eine *Good*-*Practice*-Region darstellen könnte. Im Einzelnen erreicht Westfalen-Lippe den geringsten und somit günstigsten Wert beim Versterben im Krankenhaus wie auch beim aggregierten Outcome. Die Palliativversorgungskosten, insbesondere die SAPV-Kosten, wie auch die Gesamtversorgungskosten (inkl. PV-Kosten) sind in Westfalen-Lippe pro VS auffallend gering (Ränge 6, 1 und 5, adjustiert Ränge 3, 1 und 2). Rang 1 bekleidet Westfalen-Lippe ebenfalls bei der Relation zwischen Gesamtversorgungskosten und Sterberate in der Häuslichkeit (KER). Dieses Gesamtbild ändert sich auch nach Bereinigung der Ergebnisse um Unterschiede zwischen KV-Regionen bei relevanten Patienten‑/Wohnkreismerkmalen (u. a. Grunderkrankungen, Anteil Pflegeheimpatienten, sozioökonomische Parameter) sowie bei Berücksichtigung des Anteils nicht palliativ Versorgter nicht.

In der Zusammenschau aller qualitätsbezogenen Outcomes und Kosten-Indikatoren (Tab. B.6-1) schneiden hinter Westfalen-Lippe noch Saarland, Rheinland-Pfalz und Sachsen gut ab (auch adjustiert). Hintere Ränge belegen hingegen Bremen, Bayern und Mecklenburg-Vorpommern. Die vergleichsweise günstigsten Wirkungen der PV auf die Gesamtversorgungskosten je VS weisen neben Westfalen-Lippe noch Bremen und Niedersachsen auf, die höchsten Werte Berlin und Brandenburg. Palliativversorgungskosten je VS sind in Bremen und Sachsen am geringsten, in Hamburg und Brandenburg am höchsten, mit den höchsten SAPV-Kosten je VS mit SAPV in Nordrhein bzw. Hamburg. Dass mit teuren Versorgungsmodellen (automatisch) bessere Outcomes erzielt werden, scheint auf Basis der vorliegenden Daten höchst unwahrscheinlich. In einer explorativen Regressionsanalyse über die je KV ermittelten mittleren Palliativversorgungskosten und den mittleren aggregierten Outcome (mit *n* = 17 KV-Regionen als Beobachtungen) zeigten sich keinerlei Anzeichen eines solchen Zusammenhangs (b = 0,00002; *p* = 0,908).

### Potenzielle Gründe für das vorteilhafte Abschneiden von Westfalen-Lippe

Die Gründe für das vorteilhafte Abschneiden der Palliativversorgungsregion Westfalen-Lippe sollten in zukünftigen Studien näher erforscht werden. Hierzu bieten sich auch qualitative Designs an, um ein tieferes Verständnis der palliativen Versorgungsprozesse in Westfalen-Lippe zu erhalten.

Erste Überlegungen deuten darauf hin, dass der Großteil der VS mit AAPV in Westfalen-Lippe AAPV im Rahmen der Vereinbarung gemäß §§ 37b + 132d SGB V [2] erhält, in der allgemeine und spezialisierte ambulante PV integriert angeboten werden. Teilnehmenden Hausärzten, die VS allgemein palliativmedizinisch versorgen, stehen gleichzeitig auch die Koordinationsleistungen des Palliativmedizinischen Konsiliardienstes (PKD) in Verbindung mit der Versorgungsunterstützung durch Qualifizierte Palliativärzte (QPA) – dem westfälisch-lippischen „SAPV-Modell“ – zur Verfügung. Im Gegensatz dazu gibt es in anderen KV-Regionen große Anteile an VS, die zunächst ausschließlich AAPV erhalten und bei denen SAPV später oder gar nicht hinzugezogen wird und somit nicht von Beginn an eine unterstützende spezialisierte Versorgungsstruktur wie in Westfalen-Lippe im Hintergrund verfügbar ist.

## Fazit

Unsere Studie liefert umfangreiche empirische Hinweise dafür, dass die (allgemeine und spezialisierte, ambulante und stationäre Palliativversorgung umfassende) PV in den KV-Regionen nicht nur unterschiedlich zusammengesetzt ist [5], sondern auch unterschiedliche (Ergebnis‑) Qualität/Outcomes hervorbringt und unterschiedliche Kosten erzeugt.

Die Ergebnisse deuten darauf hin, dass das Versorgungs*modell* von Westfalen-Lippe besonders vorteilhaft ist (*Good Practice*) und somit Orientierungen für andere KV-Regionen liefern könnte. Dieser Befund ist zukünftig mittels differenzierterer Betrachtungen von Qualitätsindikatoren und Patientensubgruppen sowie mittels Analysen von Daten weiterer Krankenkassen abzusichern.

Weiter ist wünschenswert, die Konstruktionsprinzipien, Voraussetzungen und Wirkmechanismen vorteilhafter Modelle sowie die Unterschiede zu regionalen Modellen mit schlechteren Outcomes herauszuarbeiten. So lassen sich möglicherweise evidenzbasierte Ansatzpunkte für eine qualitativ verbesserte und kosteneffizientere Ausgestaltung der PV in den KV-Regionen identifizieren. Deren Implementierung setzt jedoch entsprechend flexible gesetzliche Rahmenbedingungen voraus, denen der zum 01.01.2023 in Kraft getretene SAPV-Bundesrahmenvertrag gemäß § 132d Abs. 1 SGB V [41] entgegensteht. Die dort verankerten (Mindest‑)Vorgaben hinsichtlich Personalstärke und Organisation der SAPV lassen vielmehr befürchten, dass Palliativversorgungsmodelle wie das in Westfalen-Lippe entweder zu Anpassungen an den Bundesrahmenvertrag gezwungen werden oder aber bei erlaubter Koexistenz durch die höhere Leistungsvergütung in SAPV-Modellen gemäß Bundesrahmenvertrag verdrängt werden. Solche Entwicklungen sollten von der Politik sorgfältig beobachtet werden. Es sollte überprüft werden, inwieweit der neue SAPV-Bundesrahmenvertrag die empirischen Erkenntnisse aufgreifen kann, um Fehlentwicklungen zu verhindern. Patientenrelevanten Outcome-Parametern sollte stärkeres Gewicht gegeben werden als Parametern, die auf Versorgungsstrukturen abzielen. Gegebenenfalls ist mit erneuter und an empirischer Evidenz für (kosten‑)effektive Versorgungsmodelle orientierter Anpassung der gesetzlichen Rahmenbedingungen zu reagieren. Eine effiziente Erbringung von PV ist auch angesichts der demografiebedingt steigenden Sterbezahlen und eines zunehmenden Fachkräftemangels geboten.

### Supplementary Information





## Footnotes

1 Leistungen ambulanter Hospizdienste können nicht abgebildet werden, da sie nicht versichertenindividuell abgerechnet werden.
