# Supplementary material for: A regional comparison of outcomes quality and costs of general and specialized palliative care in Germany: a claims data analysis
Source: Bundesgesundheitsblatt Gesundheitsforschung Gesundheitsschutz. 2023 Aug 3;66(10):1135–45. [Article in German] doi: 10.1007/s00103-023-03746-9 (PMC10539464; doi:10.1007/s00103-023-03746-9)
Supplement: Supplementary file 1 [file 103_2023_3746_MOESM1_ESM.pdf]

Onlinematerial zum Beitrag:

Ergebnisqualität und Kosten der allgemeinen und spezialisierten Palliativversorgung in Deutschland im regionalen Vergleich: eine GKV-Routinedatenstudie

Autoren:

Antje Freytag[1], Franziska Meissner[1], Markus Krause[1], Thomas Lehmann[2], Maximiliane Katharina Jansky[3], Ursula Marschall[4], Andreas Schmid[5,6], Nils Schneider[7], Horst Christian Vollmar[8], Ulrich Wedding[9], Bianka Ditscheid[1]

[1] Institut für Allgemeinmedizin, Universitätsklinikum Jena
[2] Zentrum für Klinische Studien, Universitätsklinikum Jena
[3] Klinik für Palliativmedizin, Universitätsmedizin Göttingen
[4] BARMER Institut für Gesundheitssystemforschung, Wuppertal
[5] Gesundheitsökonomie und -management, Rechts- und Wirtschaftswissenschaftliche Fakultät, Universität Bayreuth
[6] Oberender AG, Bayreuth
[7] Institut für Allgemeinmedizin und Palliativmedizin, Medizinische Hochschule Hannover
[8] Abteilung für Allgemeinmedizin (AM RUB), Medizinische Fakultät, Ruhr-Universität Bochum
[9] Abteilung Palliativmedizin der Klinik für Innere Medizin II, Universitätsklinikum Jena

Korrespondenzadresse:

PD Dr. Antje Freytag
Institut für Allgemeinmedizin
Universitätsklinikum Jena, Friedrich-Schiller-Universität Jena
Bachstr. 18, 07743 Jena, Deutschland
Antje.Freytag@med.uni-jena.de

Inhalt

A. eMethoden: Ergänzung zur Methode 5
A.1 Datenquellen und Datenaufbereitung 5
A.2 Studienpopulation 5
A.3 Auswahl und Operationalisierung qualitätsbezogener Outcomeindikatoren 5
A.4 Bildung des aggregierten Outcomeindikators und der Ränge 7
A.5 Operationalisierung der Kostenindikatoren 8
A.6 Statistische Analysen 11
Deskriptive Analyse 11
Adjustierungsparameter der potenziell palliativversorgungsrelevanten chronischen Grunderkrankungen 11
Zur Methode der Adjustierung der Outcome- und Kostenindikatoren 11
B. eErgebnisse: Ergänzungen zu den Ergebnissen 13
B.1 Beschreibung der Studienpopulation 13
Eingeschlossene Versicherte 13
Studienpopulation nach Patienten- und Wohnkreismerkmalen 14
Zusammenhang der Adjustierungsfaktoren und ausgewählter Outcomeindikatoren 17
Inanspruchnahme der Formen von Palliativversorgung in der Studienpopulation 18
Beginn der Palliativversorgung vor dem Tod 20
B.2 Qualitätsbezogene Outcomes 21
B.3 Gesamtversorgungskosten der letzten drei Lebensmonate 24
B.4 Palliativversorgungskosten des letzten Lebensjahres 29
Palliativversorgungskosten je Versichertem mit Palliativversorgung 29
Palliativversorgungskosten je Versichertem mit der jeweiligen Palliativversorgungsform 32
B.5 Kosten-Effektivitäts-Relationen (KER) 36
B.6 Zusammenschau der Variabilität aller Zielindikatoren 39
C. eDiskussion: Ergänzungen zur Diskussion 40
C.1 Externe Validität der Outcomes 40
C.2 Externe Validität der Kosten 40
C.3 Weitere Limitationen der Studie 41
D. Literatur 42

Verzeichnis der Abbildungen

Abbildung B.1-1: Inanspruchnahme der Hauptversorgungsformen durch Versicherte mit Palliativversorgung (2016-2019) in % 18
Abbildung B.1-2: Beginn der jew. Palliativversorgungsform als Anzahl Tage vor dem Tod je KV (Mittelwert und Median) 20
Abbildung B.2-1: Qualitätsbezogene Outcomeindikatoren je KV für Versicherte mit Palliativversorgung - ADJUSTIERT 21
Abbildung B.2-2: Qualitätsbezogene Outcomeindikatoren je KV für Versicherte mit Palliativversorgung (mit 95%-Konfidenzintervall) - ADJUSTIERT 22
Abbildung B.2-3: Veränderung des aggregierten Outcomes nach Adjustierung 23
Abbildung B.3-1: Gesamtversorgungskosten der letzten drei Lebensmonate nach Leistungsbereichen je KV je Versichertem mit Palliativversorgung 27
Abbildung B.3-2: Gesamtversorgungskosten der letzten drei Lebensmonate je KV je Versichertem mit Palliativversorgung (Mittelwert (Rang), 95%-Konfidenzintervall) - ADJUSTIERT 28
Abbildung B.4-1: Palliativversorgungskosten des letzten Lebensjahres je KV je Versichertem mit Palliativversorgung (Mittelwert (Rang), 95% Konfidenzintervall) - ADJUSTIERT 31
Abbildung B.4-2: Mittelwerte ausgewählter Bestandteile der Palliativversorgungskosten im letzten Lebensjahr je KV je Versichertem mit der jeweiligen Versorgungsform (Euro) 32
Abbildung B.4-3: Mittelwerte ausgewählter Bestandteile der Palliativversorgungskosten im letzten Lebensjahr je KV je Versichertem mit der jeweiligen Versorgungsform (Euro) - ADJUSTIERT 33
Abbildung B.4-4: Mediane ausgewählter Bestandteile der Palliativversorgungskosten im letzten Lebensjahr je KV je Versichertem mit der jeweiligen Versorgungsform (Euro) 34
Abbildung B.4-5: A) SAPV-Kosten je Versichertem mit SAPV, B) SAPV-Kosten plus palliative HKP-Kosten[1] je Versichertem mit SAPV (je KV, Mittelwert (Rang), 95%-Konfidenzintervall) 35
Abbildung B.4-6: A) SAPV-Kosten je Versichertem mit SAPV, B) SAPV-Kosten plus palliative HKP-Kosten[1] je Versichertem mit SAPV (je KV, Mittelwert (Rang), 95%-Konfidenzintervall) - ADJUSTIERT 35
Abbildung B.5-1: Kosten-Effektivitäts-Relation (KER) je KV-Region - ADJUSTIERT 36

Verzeichnis der Tabellen

Tabelle A.3-1: Definition der qualitätsbezogenen Outcomeindikatoren 5
Tabelle A.5-1: Leistungsbereiche der Gesamtversorgungskosten[1] 8
Tabelle A.5-2: Bestandteile der Palliativversorgungkosten[1] 9
Tabelle A.6-1: Potenziell palliativversorgungsrelevante chronische Grunderkrankungen[1] 11
Tabelle B.1-1: Studienpopulation je KV sowie Einordnung der Studienpopulation in die Gesamtheit aller Verstorbener: Versicherte (n) und deren Anteil (%) an allen Verstorbenen sowie an der jeweiligen bundesweiten Population (alters- und geschlechtsstandardisiert) 13
Tabelle B.1-2: Patienten- und Wohnkreismerkmale, aggregiert über alle KV-Regionen (alters- und geschlechtsstandardisiert) 14
Tabelle B.1-3: Patienten- und Wohnkreismerkmale (außer Grunderkrankungen) je KV (alters- und geschlechtsstandardisiert) 15
Tabelle B.1-4: Patientenmerkmale (nur Grunderkrankungen) je KV (alters- und geschlechtsstandardisiert) 16
Tabelle B.1-5: Zusammenhang der Patienten- und Wohnkreismerkmale mit dem Outcome „Versterben im Krankenhaus“ und den Gesamtversorgungskosten der letzten drei Lebensmonate für Versicherte mit Palliativversorgung 17
Tabelle B.1-6: Inanspruchnahme der Hauptversorgungsformen durch Versicherte mit Palliativversorgung (2016-2019) in % 19
Tabelle B.3-1: Gesamtversorgungskosten sowie Pflegekosten der drei letzten Lebensmonate je KV je Versichertem mit Palliativversorgung (Euro, MW, S) 24
Tabelle B.3-2: Gesamtversorgungskosten sowie Pflegekosten der letzten drei Lebensmonate je KV je Versichertem mit Palliativversorgung (Euro, Median, IQA) 25
Tabelle B.4-1: Palliativversorgungskosten des letzten Lebensjahres je KV je Versichertem mit Palliativversorgung (Euro, MW, S) 29
Tabelle B.4-2: Palliativversorgungskosten des letzten Lebensjahres je KV je Versichertem mit Palliativversorgung (Euro, Median, IQA) 30
Tabelle B.5-1: Kosten-Effektivitäts-Relation (KER) je KV-Region 37
Tabelle B.6-1: Variabilität der qualitäts- und kostenbezogenen Outcomeindikatoren zwischen den KV-Regionen 39

A. eMethoden: Ergänzung zur Methode

A.1 Datenquellen und Datenaufbereitung

Für Details zu den Datenquellen und zur Datenaufbereitung siehe Onlinematerial (Abschnitte A.1 und A.5) zur Publikation von Ditscheid et al. 2023 [1].

A.2 Studienpopulation

In die Analysen gingen lediglich solche Verstorbenen ein, die im letzten Lebensjahr palliativ versorgt wurden, d.h. mindestens eine der folgenden Hauptformen der Hospiz- und Palliativversorgung in Anspruch genommen haben und somit als „VS mit Palliativversorgung“ gelten:

- AAPV (Allgemeine ambulante Palliativversorgung),
- BQKPmV (Besonders qualifizierte und koordinierte palliativmedizinische Versorgung),
- SAPV (Spezialisierte ambulante Palliativversorgung), der auch die durch den Palliativen Konsiliardienst (PKD) in Westfalen-Lippe geleistete Palliativversorgung zugeordnet wird,
- stationäre PV (Palliativversorgung),
- stationäre Hospizversorgung.

Für weitere versichertenbezogene Ein- und Ausschlusskriterien sowie Details zur Identifizierung der Inanspruchnahme der verschiedenen Formen von Palliativversorgung siehe Onlinematerial (Abschnitte A.2 und A.3) zur Publikation von Ditscheid et al. 2023 [1].

A.3 Auswahl und Operationalisierung qualitätsbezogener Outcomeindikatoren

Die (Ergebnis-)qualitätsbezogenen Outcomeindikatoren wurden wie folgt quantifiziert (Tabelle A.3-1).

Tabelle A.3-1: Definition der qualitätsbezogenen Outcomeindikatoren

Outcomeindikator	Bezugs-zeitraum	Definition
Versterben im Krankenhaus (Sterbeort KH)	Tod	Krankenhausfall mit Entlassungsgrund ‚079‘ (Tod)
Versterben im Krankenhaus, aber nicht auf Palliativstation (Sterbeort KH, nicht Palliativstation)	Tod	Krankenhausfall mit Entlassungsgrund ‚079‘ (Tod)<br>UND NICHT<br>Fachabteilung Entlassung ‚3752‘ (Palliativmedizin)
Rettungsdiensteinsatz	letzte 30 Lebenstage	Mind. 1 Rettungsdiensteinsatz:<br>Verordnungsart (1. Stelle des Schlüssels nach Bundeseinheitlichem Positionsnummernverzeichnis für Krankentransportleitungen)<br>…‘1‘ (Notarztwagen) ODER<br>…‘2‘ (Notarzteinsatzfahrzeug) ODER<br>…‘3‘ (Rettungswagen)
Krankenhausaufenthalt (KH-Fall)	letzte 30 Lebenstage	Mind. 1 neu beginnender, vollstationärer Krankenhausaufenthalt

**Outcomeindikator**	**Bezugs-zeitraum**	**Definition**
Krankenhausaufenthalt ohne palliativmedizinische Leistung (KH-Fall, ohne PV)	letzte 30 Lebenstage	Mind. 1 neu beginnender, vollstationärer Krankenhausaufenthalt<br>…ohne OPS 8-982*(Palliativmedizinische Komplexbehandlung) UND<br>ohne OPS 8-98e* (Spezialisierte stationäre palliativmedizinische Komplexbehandlung) UND<br>ohne OPS 8-98h* (Spezialisierte palliativmedizinische Komplexbehandlung durch einen Palliativdienst)
Intensivmedizinischer Krankenhausaufenthalt (ITS-Aufenthalt)	letzte 30 Lebenstage	Inanspruchnahme einer stationären intensivmedizinischen Behandlung:<br>§301 SGB V-Daten:<br>…OPS 8-980* (Intensivmedizinische Komplexbehandlung) ODER<br>…OPS 8-98f* (Aufwändige intensivmedizinische Komplexbehandlung)
Chemotherapie	letzte 30 Lebenstage	Inanspruchnahme mind. einer der folgenden ambulanten oder stationären Leistungen in der Teilpopulation der Versicherten mit Tumorerkrankung (ICD-10-GM C00-C97):<br>§300 SGB V-Daten:<br>PZN 09999092 (Zytostatika-Zubereitungen) ODER<br>ATC-Code L01* (Antineoplastische Mittel) UND NICHT<br>ATC-Code L01CH* (homöopathische und anthroposophische Mittel) ODER<br>ATC-Code L01CP* (pflanzliche Mittel)<br>§295 SGB V-Daten (Onkologie-Vereinbarung):<br>GOP 86514 (Zuschlag für die intrakavitär applizierte medikamentöse Tumortherapie) ODER<br>GOP 86516 (Zuschlag für die intravasal applizierte medikamentöse Tumortherapie) ODER<br>…GOP 86520 (Zuschlag für die orale medikamentöse Tumortherapie)<br>§301 SGB V-Daten:<br>OPS 8-54* (Zytostatische Chemotherapie, Immuntherapie und antiretrovirale Therapie)
Parenterale Ernährung	letzte 30 Lebenstage	Inanspruchnahme mind. einer der folgenden ambulanten oder stationären Leistungen in der Teilpopulation der Versicherten ohne gastrointestinalen Tumor ODER gastrointestinale Metastasen (ICD-10-GM: C15-C26, C78.4-C78.8):<br>§300 SGB V-Daten:<br>ATC-Code B05BA* (Lösungen zur parenteralen Ernährung) ODER<br>§301 SGB V-Daten:<br>…OPS 8-016* (Parenterale Ernährungstherapie als medizinische Hauptbehandlung) ODER<br>… OPS 8-018* (Komplette parenterale Ernährung als medizinische Nebenbehandlung)

**Outcomeindikator**	**Bezugs-zeitraum**	**Definition**
Magensonde (PEG)	letzte 30 Lebenstage	Inanspruchnahme mind. einer der folgenden ambulanten oder stationären Leistungen in der Teilpopulation der Versicherten ohne gastrointestinalen Tumor ODER gastrointestinale Metastasen (ICD-10-GM: C15-C26, C78.4-C78.8):<br>§295 SGB V-Daten:<br>GOP 13412 (Perkutane Gastrostomie) ODER<br>§301 SGB V-Daten:<br>…OPS 5-431.2* (Gastrostomie, perkutan-endoskopisch (PEG)) ODER<br>…OPS 8-123.0 (Wechsel eines Gastrostomiekatheters)

ATC-Code: Anatomisch-therapeutisch-chemischer Code, GOP: Gebührenordnungsposition des Einheitlichen Bewertungsmaßstabs, ICD-10-GM: Internationale statistische Klassifikation der Krankheiten und verwandter Gesundheitsprobleme, 10. Revision, German Modification, ITS: Intensivstation, KH: Krankenhaus, OPS: Operationen- und Prozedurenschlüssel, PEG: Perkutan-endoskopische Gastrostomie, PZN: Pharmazentralnummer

A.4 Bildung des aggregierten Outcomeindikators und der Ränge

Ergänzend wurden die neun qualitätsbezogenen Outcomeindikatoren zu einem aggregierten Outcome zusammengefasst. Hierzu wurde für alle Outcomeindikatoren zunächst die jeweilige (alters- und geschlechtsstandardisierte) mittlere Rate je KV bestimmt. Die 17 mittleren Raten jedes Outcomeindikators wurden anschließend z-standardisiert, also auf eine Skala mit einem Mittelwert von 0 und einer Standardabweichung von 1 gebracht. Der Wert für den aggregierten Outcome jeder KV wurde schließlich als arithmetisches Mittel ihrer 9 z-standardisierten Raten gebildet. Der aggregierte Outcome, in den alle neun Indikatoren mit gleichem Gewicht einfließen, misst, ob die durch diese Indikatoren geschätzte Versorgungsqualität in der betreffende KV insgesamt über dem Durchschnitt der KV-Regionen liegt (negative Werte auf dem aggregierten Outcome) oder darunter (positive Werte auf dem aggregierten Outcome). Je kleiner der Betrag des aggregierten Outcomes, desto näher liegt die betreffende KV am Durchschnitt. Umgekehrt gilt, dass ein hoher (positiver oder negativer) Wert auf dem aggregierten Outcome anzeigt, dass die betreffende KV stark vom Durchschnitt abweicht.

Zur Ausgabe des Ranges, den jede KV hinsichtlich eines bestimmten Indikators (z.B. Versterben im Krankenhaus) einnimmt, wurden die 17 KV-Regionen zunächst entsprechend ihrer Werte sortiert. Die KV mit dem qualitativ „besten“ Wert wurde dabei auf die erste Position, also auf Rang 1, gesetzt. Die anderen KV-Regionen folgen entsprechend ihrer Werte auf den dahinterliegenden Rängen. Bei einem Gleichstand mehrerer KV-Regionen, also bei exakt gleichen Werten, wird für die betreffenden KV-Regionen derselbe Rang ausgewiesen. Tritt bei einem Indikator ein solcher Fall ein, wird für die in der sortierten Verteilung nachfolgenden KV-Regionen die Rangzählung lückenlos fortgesetzt. Dies bedeutet, dass die „schlechteste“ KV nicht zwangsläufig den Rang 17 erhalten muss. Stattdessen kann der letzte Rang auch einmal Rang 16, 15, etc. sein (je nach Anzahl der beschriebenen Gleichstände in der Verteilung).

Bei nahezu allen Indikatoren, die in dieser Arbeit betrachtet werden, können niedrige Werte (d.h. niedrige Raten, niedrige Kosten) als vorteilhafter angesehen werden. Entsprechend nehmen KV-Regionen mit niedrigen Werten in der Regel die vorderen Ränge ein. Ausgenommen von dieser Regel sind der Indikator „Versterben in der Häuslichkeit“, der zur Berechnung der Kosteneffektivität

herangezogen wird, sowie die Indikatoren der Inanspruchnahme palliativer Versorgung (Inanspruchnahmeraten, Beginn der Versorgung). Bei diesen Indikatoren sind hohe Raten bzw. Werte positiv zu werten. Entsprechend wird hier jeweils der KV mit der höchsten Rate bzw. dem höchsten Wert der Rang 1 zugewiesen. Die weitere Bestimmung der Ränge folgt dem zuvor Genannten. Bei den Patientenmerkmalen wird keine Bewertung hoher/niedriger Werte vorgenommen. Ränge werden hier stets ausgehend von der höchsten Rate bzw. dem höchsten Wert vergeben.

A.5 Operationalisierung der Kostenindikatoren

Für den Vergleich der KV-Regionen hinsichtlich der Kostenwirkungen ihrer Palliativversorgung betrachteten wir die Gesamtversorgungskosten innerhalb der letzten drei Lebensmonate.

Die Gesamtversorgungskosten der letzten drei Lebensmonate summieren sich über die jeweiligen Kosten der einzelnen Leistungsbereiche (Tabelle A.5-1). Der Bezugszeitraum wurde Versicherten-individuell ausgehend vom jeweiligen Todesdatum berechnet.

Tabelle A.5-1: Leistungsbereiche der Gesamtversorgungskosten[1]

Kostenindikator	Zuordnung zum Bezugszeitraum	Definition	Anmerkung
Ambulante ärztliche Versorgung (§§294/295 SGB V, auch i.V.m. §73b, §132d und §140 SGB V)	nach Fallende	Kostenberechnung als Summe der Punktzahlen auf Fallebene, multipliziert mit dem jeweilig gültigen Orientierungspunktwert und Addition weiterer Kosten, die mit Kostenwert angegeben sind (Selektivvertragliche Leistungen).	Behandlungsfälle gehen als Ganzes ein; ggf. sind dies zwei Quartale → Überschätzung dieser Kosten; enthalten sind hier auch die Kosten der AAPV, BQKPMV sowie SAPV-Verordnungen, selektivvertragliche, ärztliche SAPV/PKD[2]–Leistungen (Berlin, Nordrhein und Westfalen-Lippe).
HKP (§302 SGB V, auch i.V.m. §132d SGB V)	anteilig nach Leistungs-zeitraum	Summe aller HKP-Leistungen im Bezugszeitraum.	Enthalten auch SAPV-Leistungsabrechnung-en.
Krankenhaus (§ 301 SGB V)	anteilig nach Leistungs-zeitraum	Summe der Krankenhausfallkosten im Bezugszeitraum abzgl. Zuzahlung.	Umfasst voll-, teil- und vorstationäre Krankenhaus-behandlungen, auch im Krankenhaus erbrachte palliativmedizinische Leistungen.
Rettungsdienst (§302 SGB V)	nach Leistungs-beginn	Nettosumme der Kosten aller Rettungsdiensteinsätze im Bezugszeitraum.	Ggf. angefallene Zuzahlungen sind berücksichtigt.
Krankentransport (§302 SGB V)	nach Leistungs-beginn	Nettosumme der Kosten aller Fahrten und Krankentransporte im Bezugszeitraum.	Ggf. angefallene Zuzahlungen sind berücksichtigt.

Kostenindikator	Zuordnung zum Bezugszeitraum	Definition	Anmerkung
Hospiz (§39a SGB V)	anteilig nach Leistungs-zeitraum	Summe aller Zuschüsse für stationäre, teilstationäre und ambulante Hospizbehandlungen im Bezugszeitraum.[3]	
Arzneimittel (§300 SGB V)	nach dem Arzneimittel-Abgabedatum	Bruttosumme der Kosten aller Arzneimittel im Bezugszeitraum.	Über die Apotheke abgegebene Hilfsmittel sind ebenfalls enthalten. Ggf. angefallene Zuzahlungen und Rabatte sind nicht berücksichtigt.
Heilmittel (§302 SGB V)	anteilig nach Leistungs-zeitraum	Nettosumme der Kosten aller Heilmittel im Bezugszeitraum.	Ggf. angefallene Zuzahlungen sind berücksichtigt.
Hilfsmittel (§302 SGB V)	anteilig nach Leistungs-zeitraum	Nettosumme der Kosten aller Hilfsmittel im Bezugszeitraum.	Ggf. angefallene Zuzahlungen sind berücksichtigt.
Pflegeleistungen (SGB XI) *separat ausgewiesen*	anteilig nach Rechnungs-zeitraum	Summe der Kosten aller Pflege- und Zusatzleistungen im Bezugszeitraum.	Nur für die Subpopulation der Verstorbenen aus 2018 und 2019 verfügbar.

HKP: Häusliche Krankenpflege (SGB V),
[1]Bezugszeitraum: letzte 3 Lebensmonate, [2](ambulanter) Palliativmedizinischer Konsiliardienst in Westfalen-Lippe, [3]zu 99,5% stationäre Hospizbehandlungen

Während die Kostenwirkungen der Palliativversorgung in einer KV-Region also anhand der Gesamtversorgungskosten der letzten drei Lebensmonate gemessen wurden, die auch die Palliativversorgungskosten in diesem Zeitraum enthalten, verglichen wir darüber hinaus explizit auch die Kosten der Palliativversorgung („Interventionskosten“) zwischen den KV-Regionen. Äquivalent zur Einschlussbedingung der Studienpopulation „Verstorbene mit Palliativversorgung im letzten Lebensjahr“, bezogen wir dazu auch die Palliativversorgungskosten des gesamten letzten Lebensjahres ein.

Tabelle A.5-2 erläutert die Kostenbestandteile der Palliativversorgungskosten des letzten Lebensjahres.

Die jeweils zugrundeliegenden Einzelleistungen und deren Identifizierung in den GKV-Routinedaten wurden bereits ausführlich im Onlinematerial unserer vorausgegangenen Publikation durch Ditscheid et al. 2023 [1] beschrieben.

Tabelle A.5-2: Bestandteile der Palliativversorgungkosten[1]

Kostenindikator	Zuordnung zum Bezugszeitraum	Definition
Bestandteile der Palliativversorgungskosten		
AAPV	nach dem Leistungsdatum	Kostenberechnung als Summe der Punktzahlen für alle im Bezugszeitraum abgerechneten Leistungen multipliziert mit dem jeweils gültigen Orientierungspunktwert und Addition von Kosten für

**Kostenindikator**	**Zuordnung zum Bezugszeitraum**	**Definition**
		AAPV-Leistungen aus HzV- und Selektivverträgen, einschließlich der Onkologie-Vereinbarung (16) im Bezugszeitraum.<br>Zu den (erweiterten) AAPV-Leistungen zählen wir auch die von den Qualifizierten Palliativärzten (QPA) in Nordrhein abgerechneten Leistungen der Palliativversorgung.
BQKPmV	nach dem Leistungsdatum	Kostenberechnung als Summe der Punktzahlen für alle im Bezugszeitraum abgerechneten Leistungen multipliziert mit dem jeweils gültigen Orientierungspunktwert
SAPV	nach dem Leistungsdatum (SAPV-Verordnung, SAPV aus Selektivverträgen) bzw. anteilig nach Leistungszeitraum (SAPV aus HKP-Daten)	Kostenberechnung als Summe der Punktzahlen für alle im Bezugszeitraum abgerechneten Leistungen multipliziert mit dem jeweils gültigen Orientierungspunktwert (SAPV-Verordnungen), Addition von Kosten für alle SAPV-Leistungen aus Selektivverträgen im Bezugszeitraum und von Kosten für SAPV-Leistungen aus HKP-Daten im Bezugszeitraum.<br>Zur SAPV zählen wir auch die Leistungen der Palliativmedizinischen Konsiliardienste (PKD) in Verbindung mit den Qualifizierten Palliativärzten (QPA) in Westfalen-Lippe.
Palliative HKP (HKPpall)	anteilig nach Leistungs-zeitraum	Summe aller palliativen HKP-Leistungen im Bezugszeitraum
HKP ab erster ambulanter Palliativleistung (HKP_aP)	nach dem Leistungsbeginn	Summe aller palliativen HKP-Leistungen, deren Leistungsbeginn ≥ dem Leistungsdatum der ersten ambulanten Palliativleistung ist
Stationäre Palliativversorgung im Krankenhaus (stat.PV)	anteilig nach Leistungs-zeitraum	Summe der Entgeltkosten für palliative OPS und palliative Entgelte im Bezugszeitraum.
Hospiz	anteilig nach Leistungs-zeitraum	Summe aller Zuschüsse für stationäre, teilstationäre und ambulante Hospizbehandlungen im Bezugszeitraum.[2]
Zusätzlich ausgewiesen:		
HKP gesamt	anteilig nach Leistungs-zeitraum	Summe aller HKP-Leistungen im Bezugszeitraum (abzüglich der in diesem Leistungsbereich enthaltenen SAPV-Leistungen)

AAPV: Allgemeine ambulante Palliativversorgung, BQKPmV: Besonders qualifizierte und koordinierte palliativmedizinische Versorgung, HKP: Häusliche Krankenpflege (SGB V), OPS: Operationen- und Prozedurenschlüssel, PV: Palliativversorgung, SAPV: Spezialisierte ambulante Palliativversorgung
[1]Bezugszeitraum: letztes Lebensjahr, [2]zu 99,5% stationäre Hospizbehandlungen

A.6 Statistische Analysen

Die Analysen führten wir mit R, Version 4.1.2, durch. Alle Analysen beruhen auf alters- und geschlechtsstandardisierten Daten. Die zur Standardisierung herangezogenen Gewichte waren im Rahmen einer früheren Arbeit basierend auf öffentlichen Todesfallstatistiken je Jahr und Bundesland ermittelt worden [1] und wurden für die aktuelle Arbeit übernommen. Die Standardisierungsfaktoren bezogen wir in allen Analysen als Gewichte ein und nutzten dafür Funktionen aus dem R Package *survey* [2]. Für eine Erläuterung zur Ermittlung der Standardisierungsfaktoren sei auf das Onlinematerial von Ditscheid et al. (2013) verwiesen [1].

Um eine ausreichende Datenbasis für die zentralen Zielgrößen zu gewährleisten, erfolgt keine Analyse der Zielgrößen nach dem Sterbejahr der VS.

Deskriptive Analyse

Für die deskriptive Analyse der Patientenmerkmale, Outcomeindikatoren und Kosten berechneten wir je nach Skalenniveau der Variablen den Anteil in % bzw. Mittelwert und Standardabweichung sowie für ausgewählte Größen weitere statistische Kenngrößen (Median, Min, Max, etc.).

Adjustierungsparameter der potenziell palliativversorgungsrelevanten chronischen Grunderkrankungen

Die Liste der ICD-10-Diagnosen für die Identifikation potenziell palliativversorgungsrelevanter chronischer Grunderkrankungen (Tabelle A.6-1) orientiert sich an Murtagh et al. [3].

Tabelle A.6-1: Potenziell palliativversorgungsrelevante chronische Grunderkrankungen[1]

Grunderkrankung	ICD-10-GM Codes
Tumorerkrankung	C00*-C97*
Herz-Kreislauf-Erkrankung	I0*-I6*
Herzinsuffizienz	I50*
Zerebrovaskuläre Erkrankung	I60*-I69*
Nierenerkrankung	N17*-N18*, N28*, I12*-I13*
Lebererkrankung	K70*-K77*
Atemwegserkrankung	J09*-J18*, J20*-J22*, J40*-J47*, J96*
Neurodegenerative Erkrankung	G10*, G20*, G35*, G122*, G231-G233*
Demenz, Alzheimer, Senilität	F00*-F03*, G30*, R54*
HIV, AIDS	B20*-B24*

[1]*identifiziert anhand einer mindestens einmalig kodierten Diagnose (gesichert ambulant, Krankenhaushaupt- oder -nebendiagnose) im letzten Lebensjahr*

Zur Methode der Adjustierung der Outcome- und Kostenindikatoren

Ausgegeben wurden die *Marginal Predictive Means*, also die Raten/Kosten, die sich rechnerisch ergeben, wenn in allen KV-Regionen die gleiche (nämlich die über die gesamte Studienpopulation hinweg beobachtete) Verteilung der Patienten- und Wohnkreismerkmale angenommen wird. Diese Methode der Berechnung (*marginal standardization*) ist in diesem Fall anderen Methoden der Bestimmung adjustierter Werte vorzuziehen [4]. Für die Berechnung nutzten wir die Funktion *svypredmeans* aus dem R Package *survey* [2]. Grundlage der Berechnung ist bei den binären Outcomeindikatoren ein multiples logistisches, bei den Kostenindikatoren ein multiples lineares Regressionsmodell (OLS Regression). Obwohl das Survey-Package robuste Standardfehler garantiert, sicherten wir unsere Schlussfolgerungen hinsichtlich der Kostenindikatoren gegen eine Verzerrung aufgrund der Schiefe der Verteilung ab. Hierzu wurde die Analyse auf Basis eines Generalized Linear Models (GLM) mit Gamma-Verteilung und Log-Link wiederholt. Die im Manuskript berichteten

Schlussfolgerungen ändern sich dadurch nicht, weshalb wir die leichter zugänglichen Ergebnisse auf Basis des linearen Regressionsmodells berichten.

B. eErgebnisse: Ergänzungen zu den Ergebnissen

B.1 Beschreibung der Studienpopulation

Eingeschlossene Versicherte

Tabelle B.1-1: Studienpopulation je KV sowie Einordnung der Studienpopulation in die Gesamtheit aller Verstorbener: Versicherte (n) und deren Anteil (%) an allen Verstorbenen sowie an der jeweiligen bundesweiten Population (alters- und geschlechtsstandardisiert)

	**Alle Verstorbenen** (N = 417.405)			**Studienpopulation: Verstorbene mit PV** (N = 145.372)				**Verstorbene ohne PV** (N = 272.033)		
		**Anteil an allen VS**	**Anteil an Dtl.**		**Anteil an allen VS**		**Anteil an Dtl.**		**Anteil an allen VS**	**Anteil an Dtl.**
	**n**	**%**	**%**	**n**	**%**	**Rang**	**%**	**n**	**%**	**%**
**KV**										
Baden-Württemberg	34.884	100,0	8,4	**11.597**	**33,2**	**11**	**8,0**	23.287	66,8	8,6
Bayern	52.092	100,0	12,5	**23.279**	**44,7**	**1**	**16,0**	28.813	55,3	10,6
Berlin	19.683	100,0	4,7	**6.603**	**33,5**	**10**	**4,5**	13.080	66,5	4,8
Brandenburg	15.701	100,0	3,8	**4.931**	**31,4**	**14**	**3,4**	10.770	68,6	4,0
Bremen	1.955	100,0	0,5	**548**	**28,0**	**17**	**0,4**	1.407	72,0	0,5
Hamburg	8.678	100,0	2,1	**2.838**	**32,7**	**13**	**2,0**	5.840	67,3	2,1
Hessen	35.675	100,0	8,5	**12.250**	**34,3**	**6**	**8,4**	23.425	65,7	8,6
Mecklenburg-Vorpommern	9.770	100,0	2,3	**3.377**	**34,6**	**5**	**2,3**	6.393	65,4	2,4
Niedersachsen	39.842	100,0	9,5	**14.302**	**35,9**	**2**	**9,8**	25.540	64,1	9,4
Nordrhein	63.461	100,0	15,2	**21.322**	**33,6**	**9**	**14,7**	42.139	66,4	15,5
Rheinland-Pfalz	21.595	100,0	5,2	**7.466**	**34,6**	**4**	**5,1**	14.129	65,4	5,2
Saarland	5.219	100,0	1,3	**1.777**	**34,1**	**7**	**1,2**	3.442	65,9	1,3
Sachsen	20.185	100,0	4,8	**6.148**	**30,5**	**15**	**4,2**	14.037	69,5	5,2
Sachsen-Anhalt	12.220	100,0	2,9	**3.450**	**28,2**	**16**	**2,4**	8.770	71,8	3,2
Schleswig-Holstein	19.053	100,0	4,6	**6.411**	**33,6**	**8**	**4,4**	12.642	66,4	4,6
Thüringen	11.098	100,0	2,7	**3.936**	**35,5**	**3**	**2,7**	7.162	64,5	2,6
Westfalen-Lippe	46.294	100,0	11,1	**15.138**	**32,7**	**12**	**10,4**	31.156	67,3	11,5
**Deutschland**	**417.405**	**100,0**	**100,0**	**145.372**	**34,8**		**100,0**	**272.033**	**65,2**	**100,0**

KV: Kassenärztliche Vereinigung, PV: Palliativversorgung, VS: Verstorbene. Als Studienpopulation (Mitte, fett hervorgehoben) wurden in dieser Arbeit ausschließlich Verstorbene mit PV im letzten Lebensjahr betrachtet.

Studienpopulation nach Patienten- und Wohnkreismerkmalen

Tabelle B.1-2: Patienten- und Wohnkreismerkmale, aggregiert über alle KV-Regionen (alters- und geschlechtsstandardisiert)

	**N = 145.372**
Geschlecht (weiblich), n (%)	77.551 (53,3%)
Alter, MW (S)	78,59 (12,3)
CCI, MW (S)	8,31 (4,1)
Tumor, n (%)	94.886 (65,3%)
Herz-Kreislauf-Erkrankung, n (%)	131.743 (90,6%)
Herzinsuffizienz, n (%)	59.802 (41,1%)
Zerebrovaskuläre Erkrankung, n (%)	47.656 (32,8%)
Nierenerkrankung, n (%)	71.580 (49,2%)
Lebererkrankung, n (%)	31.279 (21,5%)
Atemwegserkrankung, n (%)	87.040 (59,9%)
Neurodegenerative Erkrankung, n (%)	11.297 (7,8%)
Demenz, Alzheimer, Senilität, n (%)	66.588 (45,8%)
HIV, AIDS, n (%)	202 (0,1%)
Pflegebedarf, n (%)	
Häuslichkeit ohne Pflegegrad	16.480 (11,3%)
Häuslichkeit mit Pflegegrad	102.523 (70,5%)
Pflegeheim	26.368 (18,1%)
Deprivationsgrad, MW (S)	0,52 (0,2)
Ländlichkeit, MW (S)	0,19 (0,3)

CCI: Charlson Comorbidity Index, GE: Grunderkrankung, KV: Kassenärztliche Vereinigung, MW: Mittelwert, S: Standardabweichung

Tabelle B.1-3: Patienten- und Wohnkreismerkmale (außer Grunderkrankungen) je KV (alters- und geschlechtsstandardisiert)

	Geschlecht (weiblich)		Alter		CCI		Pflegebedarf: Häuslichkeit ohne Pflegegrad		Pflegebedarf: Häuslichkeit mit Pflegegrad		Pflegebedarf: Pflegeheim		Deprivationsgrad		Ländlichkeit	
KV	%	Rang	MW (S)	Rang	MW (S)	Rang	%	Rang	%	Rang	%	Rang	MW (S)	Rang	MW (S)	Rang
Baden-Württemberg	53,8	5	79,08 (12,4)	2	8,00 (4,1)	14	10,6	9	70,7	11	18,7	4	0,40 (0,1)	15	0,10 (0,2)	11
Bayern	54,5	2	80,05 (12,1)	1	7,99 (4,1)	15	15,6	1	63,0	17	21,4	1	0,33 (0,2)	16	0,27 (0,3)	8
Berlin	53,6	6	77,30 (12,4)	14	9,08 (4,0)	2	12,6	4	70,0	14	17,4	7	0,58 (0,0)	11	0,00 (0,0)	15
Brandenburg	49,4	17	76,57 (12,3)	17	9,31 (4,1)	1	10,6	10	76,6	3	12,8	16	0,63 (0,1)	6	0,41 (0,3)	3
Bremen	54,2	3	78,40 (11,6)	7	7,89 (4,0)	16	9,9	12	75,4	5	14,7	12	0,65 (0,1)	3	0,00 (0,0)	15
Hamburg	52,2	11	77,32 (12,6)	13	8,31 (4,0)	10	14,4	2	68,4	16	17,1	9	0,32 (0,0)	17	0,00 (0,0)	15
Hessen	53,5	7	78,86 (12,4)	3	7,88 (4,0)	17	10,5	11	72,3	8	17,2	8	0,43 (0,1)	14	0,15 (0,2)	10
Mecklenburg-Vorpommern	51,2	14	77,01 (11,8)	16	8,92 (4,0)	5	8,4	17	78,9	1	12,7	17	0,72 (0,0)	2	0,54 (0,3)	1
Niedersachsen	53,1	8	78,65 (12,2)	5	8,19 (4,1)	11	9,3	14	70,4	13	20,3	2	0,57 (0,1)	12	0,29 (0,2)	7
Nordrhein	54,9	1	78,72 (12,2)	4	8,17 (4,1)	12	9,0	16	72,0	10	19,1	3	0,57 (0,2)	13	0,02 (0,1)	14
Rheinland-Pfalz	52,9	9	78,42 (12,1)	6	8,31 (4,1)	9	12,6	5	70,5	12	16,9	10	0,60 (0,1)	10	0,30 (0,3)	6
Saarland	52,6	10	77,95 (11,8)	11	8,60 (4,1)	7	11,6	7	75,6	4	12,8	15	0,64 (0,1)	5	0,06 (0,1)	13
Sachsen	51,9	12	78,24 (12,0)	9	8,82 (4,1)	6	12,4	6	73,0	7	14,6	13	0,61 (0,1)	8	0,22 (0,2)	9
Sachsen-Anhalt	50,2	16	77,15 (11,8)	15	9,07 (4,1)	3	9,1	15	77,1	2	13,8	14	0,76 (0,1)	1	0,42 (0,3)	2
Schleswig-Holstein	51,8	13	77,75 (12,3)	12	8,02 (4,0)	13	13,3	3	68,8	15	18,0	6	0,62 (0,1)	7	0,32 (0,3)	5
Thüringen	51,0	15	78,15 (11,6)	10	9,00 (4,1)	4	10,8	8	73,8	6	15,3	11	0,65 (0,1)	4	0,37 (0,3)	4
Westfalen-Lippe	54,0	4	78,39 (12,5)	8	8,43 (4,0)	8	9,7	13	72,1	9	18,2	5	0,60 (0,1)	9	0,09 (0,1)	12
**Deutschland**	**53,3**	-	**78,59 (12,3)**	-	**8,31 (4,1)**	-	**11,3**	-	**70,5**	-	**18,1**	-	**0,52 (0,2)**	-	**0,19 (0,3)**	-

CCI: Charlson Comorbidity Index, KV: Kassenärztliche Vereinigung, MW: Mittelwert, S: Standardabweichung

Tabelle B.1-4: Patientenmerkmale (nur Grunderkrankungen) je KV (alters- und geschlechtsstandardisiert)

	**Tumor**		**Herz-Kreislauf-Erkrankung**		**Herzinsuffizienz**		**Zerebrovasku-läre Erkrankung**		**Nieren-erkrankung**		**Leber-erkrankung**		**Atemwegs-erkrankung**		**Neurodegenera-tive Erkrankung**		**Demenz, Alzheimer, Senilität**		**HIV, AIDS**	
**KV**	**%**	**Rang**	**%**	**Rang**	**%**	**Rang**	**%**	**Rang**	**%**	**Rang**	**%**	**Rang**	**%**	**Rang**	**%**	**Rang**	**%**	**Rang**	**%**	**Rang**
Baden-Württemberg	63,9	16	89,3	14	38,6	13	32,1	11	46,7	11	22,0	8	58,7	10	7,3	9	51,7	2	0,1	9
Bayern	57,9	17	91,2	7	45,9	2	34,8	2	53,7	5	23,6	4	60,8	7	8,1	5	49,3	3	0,1	11
Berlin	72,0	4	89,6	13	39,8	9	29,7	14	56,1	3	22,3	7	61,2	6	7,1	13	43,2	8	0,3	1
Brandenburg	74,8	1	91,3	6	37,2	14	28,5	16	59,1	2	22,8	5	56,8	14	6,4	17	36,1	16	0,0	15
Bremen	69,3	7	87,9	15	32,4	17	26,6	17	37,1	17	21,6	9	54,3	16	7,6	8	35,8	17	0,0	16
Hamburg	72,0	3	86,4	17	34,1	16	30,2	12	41,2	16	17,9	16	59,3	9	7,2	11	45,1	5	0,3	2
Hessen	64,3	14	89,9	12	38,7	12	30,2	13	44,0	14	20,1	14	57,5	13	8,0	6	40,3	12	0,1	8
Mecklenburg-Vorpommern	72,0	5	92,1	4	39,7	10	32,5	9	53,8	4	22,7	6	58,6	11	6,7	15	41,9	11	0,1	7
Niedersachsen	64,3	13	90,4	10	42,9	6	32,7	8	47,1	10	20,0	15	59,6	8	7,2	12	54,4	1	0,1	12
Nordrhein	64,6	12	90,9	9	39,2	11	33,9	5	45,9	12	21,2	11	61,4	5	8,4	3	48,3	4	0,2	4
Rheinland-Pfalz	64,2	15	91,8	5	43,1	5	32,9	7	47,3	9	21,5	10	64,2	1	8,2	4	43,0	9	0,2	3
Saarland	67,7	9	91,1	8	40,0	8	38,2	1	43,7	15	25,7	1	61,7	4	8,9	1	42,8	10	0,2	5
Sachsen	69,6	6	93,0	2	43,4	4	33,5	6	53,1	6	20,5	13	52,8	17	6,5	16	40,0	13	0,1	13
Sachsen-Anhalt	72,1	2	92,7	3	43,5	3	32,4	10	53,1	7	24,2	2	56,3	15	7,3	10	36,8	15	0,0	14
Schleswig-Holstein	68,7	8	87,4	16	35,3	15	28,9	15	44,7	13	17,4	17	58,3	12	8,0	7	38,0	14	0,1	6
Thüringen	67,1	10	93,9	1	46,9	1	34,3	4	60,7	1	23,9	3	61,9	2	7,1	14	43,9	7	0,0	16
Westfalen-Lippe	66,3	11	90,3	11	41,2	7	34,6	3	48,3	8	21,0	12	61,9	3	8,6	2	44,4	6	0,1	10
**Deutschland**	**65,3**	**-**	**90,6**	**-**	**41,1**	**-**	**32,8**	**-**	**49,2**	**-**	**21,5**	**-**	**59,9**	**-**	**7,8**	**-**	**45,8**	**-**	**0,1**	**-**

KV: Kassenärztliche Vereinigung

Zusammenhang der Adjustierungsfaktoren und ausgewählter Outcomeindikatoren

Tabelle B.1-5: Zusammenhang der Patienten- und Wohnkreismerkmale mit dem Outcome „Versterben im Krankenhaus“ und den Gesamtversorgungskosten der letzten drei Lebensmonate für Versicherte mit Palliativversorgung

	Sterbeort Krankenhaus		Gesamtversorgungskosten (letzte 3 Lebensmonate, ohne Pflegekosten)	
	**OR**	**p**	**B**	**p**
Geschlecht: weiblich	0,68	***	-3.754	***
Alter	0,96	***	-494	***
CCI	1,11	***	931	***
Tumor	2,20	***	7.392	***
Herz-Kreislauf-Erkrankung	0,92	***	-2.705	***
Herzinsuffizienz	1,09	***	-1.289	***
Zerebrovaskuläre Erkrankung	0,83	***	-1.470	***
Nierenerkrankung	1,53	***	2.002	***
Lebererkrankung	1,67	***	3.757	***
Atemwegserkrankung	1,98	***	4.172	***
Neurodegenerative Erkrankung	0,59	***	-3.587	***
Demenz, Alzheimer, Senilität	0,41	***	-9.023	***
HIV, AIDS	1,60	**	10.131	***
Pflegebedarf: Häuslichkeit mit Pflegegrad	0,11	***	-3.413	***
Pflegebedarf: Pflegeheim	0,04	***	-13.751	***
Deprivationsgrad	0,77	***	1.791	***
Ländlichkeit	1,11	***	171	ns

*Ergebnisse beruhen auf einfachen logistischen (Spalte: Sterbeort Krankenhaus) bzw. linearen (Spalte: Gesamtversorgungskosten) Regressionsmodellen mit der in der Spalte genannten Variable als Outcome und der in der Zeile genannten Variable als einzigem Prädiktor. Ausgewiesen werden Odds Ratios (OR) bzw. Regressionsgewichte (B) sowie die zugehörige Signifikanz (p). *** p < 0,001; ** p < 0,01; * p < 0,05; ns: nicht signifikant. CCC: Charlson Comorbidity Index*

Inanspruchnahme der Formen von Palliativversorgung in der Studienpopulation

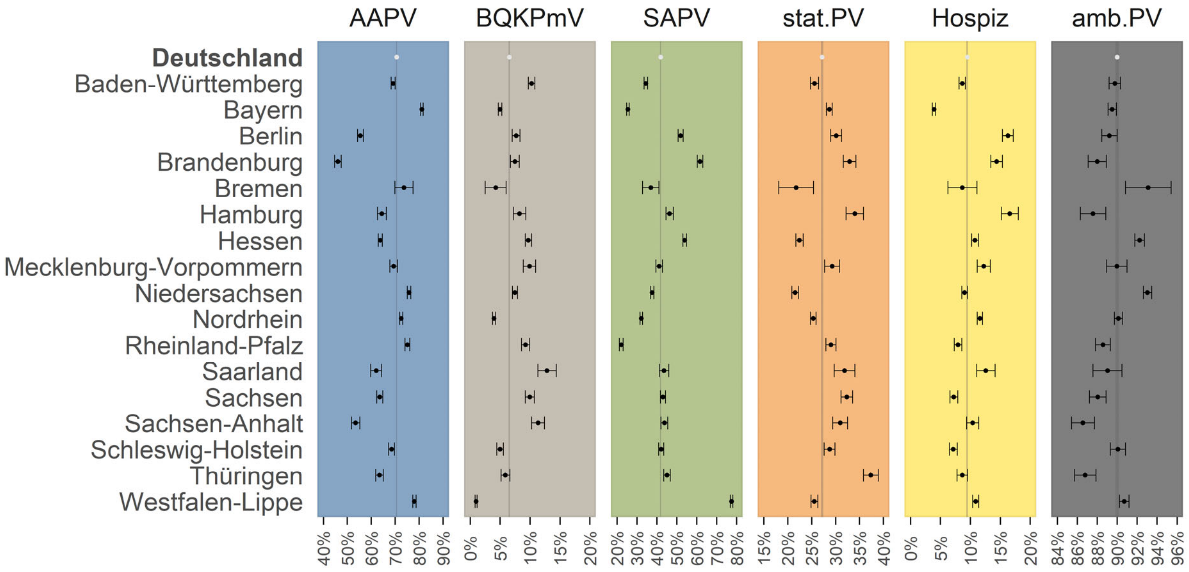

Abbildung B.1-1: Inanspruchnahme der Hauptversorgungsformen durch Versicherte mit Palliativversorgung (2016-2019) in %

AAPV: Allgemeine ambulante Palliativversorgung, amb.PV: Ambulante Palliativversorgung, BQKPmV: Besonders qualifizierte und koordinierte palliativmedizinische Versorgung, PV: Palliativversorgung, SAPV: Spezialisierte ambulante Palliativversorgung, stat.PV: Stationäre Palliativversorgung im Krankenhaus

Die Inanspruchnahmeraten je KV (+95%-Konfidenzintervall) sind als schwarze Punkte (+Whisker) dargestellt. Als Referenz wird außerdem die Inanspruchnahmerate über alle Versicherte mit Palliativversorgung gezeigt („Deutschland“); die Breite dieser Referenzlinie repräsentiert ihr Konfidenzintervall.

Quelle: eigene Abbildung

Tabelle B.1-6: Inanspruchnahme der Hauptversorgungsformen durch Versicherte mit Palliativversorgung (2016-2019) in %

	AAPV		BQKPmV		SAPV		stat.PV		Hospiz		amb.PV	
**KV**	**p (CI)**	**Rang**	**p (CI)**	**Rang**	**p (CI)**	**Rang**	**p (CI)**	**Rang**	**p (CI)**	**Rang**	**p (CI)**	**Rang**
Baden-Württemberg	69,1 (68,3 - 70,0)	8	10,2 (9,7 - 10,8)	3	34,4 (33,5 - 35,3)	14	25,6 (24,8 - 26,4)	12	8,6 (8,1 - 9,2)	13	89,8 (89,2 - 90,3)	8
Bayern	81,1 (80,6 - 81,6)	1	5,0 (4,7 - 5,3)	14	25,5 (24,9 - 26,0)	16	28,7 (28,1 - 29,3)	11	3,9 (3,7 - 4,2)	17	89,5 (89,1 - 89,9)	9
Berlin	55,5 (54,3 - 56,7)	15	7,7 (7,0 - 8,3)	9	51,9 (50,7 - 53,1)	4	30,1 (29,0 - 31,2)	7	16,3 (15,4 - 17,2)	2	89,2 (88,4 - 90,0)	10
Brandenburg	46,1 (44,6 - 47,5)	17	7,5 (6,7 - 8,2)	11	61,6 (60,2 - 63,0)	2	32,9 (31,6 - 34,3)	3	14,4 (13,4 - 15,3)	3	88,0 (87,1 - 88,9)	14
Bremen	73,7 (69,8 - 77,5)	5	4,3 (2,5 - 6,0)	15	36,9 (32,8 - 41,1)	13	21,8 (18,1 - 25,4)	16	8,7 (6,2 - 11,1)	11	93,1 (90,8 - 95,4)	1
Hamburg	64,4 (62,6 - 66,2)	10	8,3 (7,2 - 9,3)	8	46,4 (44,5 - 48,3)	5	34,0 (32,2 - 35,8)	2	16,6 (15,2 - 18,0)	1	87,6 (86,3 - 88,8)	15
Hessen	63,7 (62,9 - 64,6)	11	9,7 (9,2 - 10,2)	6	54,0 (53,1 - 54,9)	3	22,5 (21,7 - 23,2)	15	10,8 (10,2 - 11,3)	8	92,2 (91,8 - 92,7)	3
Mecklenburg-Vorpommern	69,3 (67,7 - 70,9)	7	9,9 (8,9 - 10,9)	5	41,2 (39,5 - 42,9)	11	29,3 (27,7 - 30,8)	8	12,2 (11,1 - 13,3)	5	90,0 (88,9 - 91,0)	7
Niedersachsen	75,8 (75,0 - 76,5)	3	7,5 (7,1 - 7,9)	10	37,6 (36,8 - 38,5)	12	21,5 (20,8 - 22,2)	17	9,0 (8,6 - 9,5)	10	93,0 (92,6 - 93,5)	2
Nordrhein	72,5 (71,9 - 73,1)	6	4,0 (3,7 - 4,2)	16	32,1 (31,5 - 32,8)	15	25,3 (24,7 - 25,9)	14	11,6 (11,1 - 12,0)	6	90,1 (89,7 - 90,5)	5
Rheinland-Pfalz	75,1 (74,1 - 76,1)	4	9,2 (8,6 - 9,9)	7	22,2 (21,2 - 23,1)	17	29,0 (28,0 - 30,1)	9	7,9 (7,3 - 8,6)	14	88,6 (87,8 - 89,3)	12
Saarland	62,0 (59,8 - 64,3)	14	12,8 (11,3 - 14,4)	1	43,6 (41,2 - 45,9)	8	31,9 (29,7 - 34,0)	5	12,6 (11,0 - 14,1)	4	89,0 (87,6 - 90,5)	11
Sachsen	63,5 (62,3 - 64,8)	12	10,0 (9,2 - 10,7)	4	43,0 (41,8 - 44,3)	9	32,3 (31,1 - 33,5)	4	7,2 (6,6 - 7,9)	15	88,0 (87,2 - 88,9)	13
Sachsen-Anhalt	53,5 (51,8 - 55,2)	16	11,3 (10,3 - 12,4)	2	43,8 (42,1 - 45,5)	7	31,0 (29,4 - 32,5)	6	10,4 (9,4 - 11,4)	9	86,6 (85,4 - 87,7)	17
Schleswig-Holstein	68,4 (67,3 - 69,6)	9	5,0 (4,5 - 5,5)	13	42,2 (40,9 - 43,4)	10	28,8 (27,6 - 29,9)	10	7,1 (6,5 - 7,8)	16	90,1 (89,3 - 90,8)	6
Thüringen	63,5 (61,9 - 65,0)	13	5,9 (5,1 - 6,6)	12	45,1 (43,5 - 46,7)	6	37,4 (35,8 - 38,9)	1	8,6 (7,8 - 9,5)	12	86,8 (85,7 - 87,9)	16
Westfalen-Lippe	78,0 (77,3 - 78,7)	2	1,0 (0,8 - 1,1)	17	77,4 (76,7 - 78,1)	1	25,6 (24,9 - 26,3)	13	10,9 (10,4 - 11,4)	7	90,7 (90,2 - 91,2)	4
**Deutschland**	**70,6 (70,4 - 70,9)**	**-**	**6,6 (6,4 - 6,7)**	**-**	**41,9 (41,7 - 42,2)**	**-**	**27,2 (27,0 - 27,4)**	**-**	**9,5 (9,3 - 9,6)**	**-**	**90,0 (89,8 - 90,2)**	**-**

AAPV: Allgemeine ambulante Palliativversorgung, amb.PV: Ambulante Palliativversorgung (AAPV, BQKPmV, SAPV), BQKPmV: Besonders qualifizierte und koordinierte palliativmedizinische Versorgung, KV: Kassenärztliche Vereinigung, SAPV: Spezialisierte ambulante Palliativversorgung, stat.PV: Stationäre Palliativversorgung im Krankenhaus

Beginn der Palliativversorgung vor dem Tod

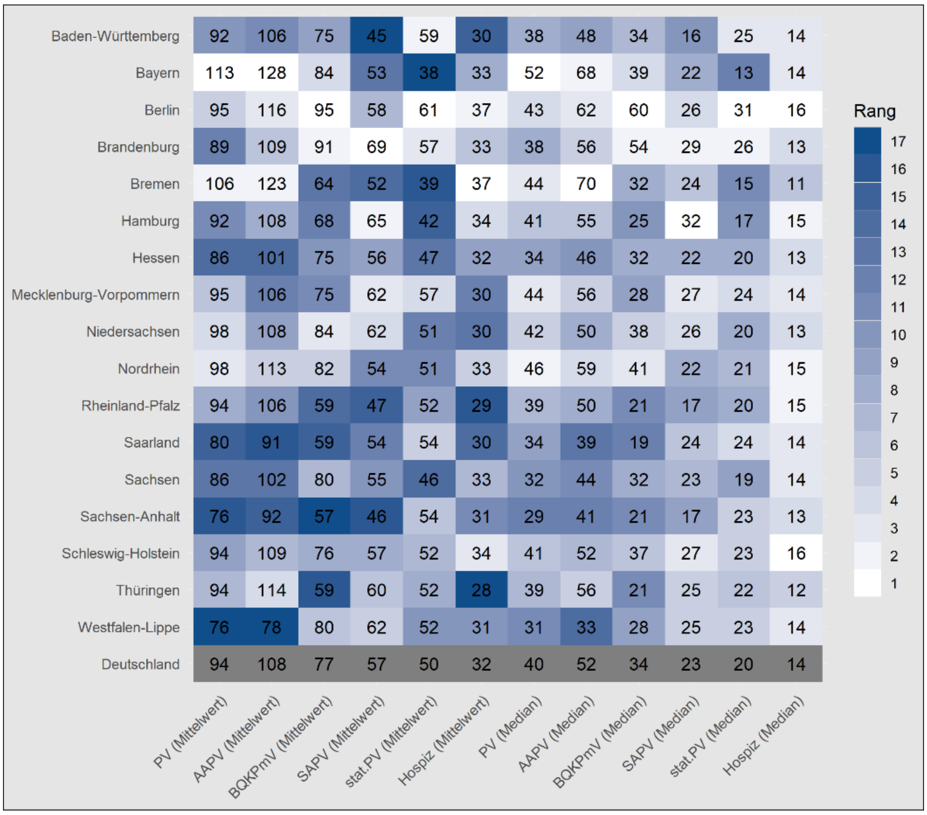

Abbildung B.1-2: Beginn der jew. Palliativversorgungsform als Anzahl Tage vor dem Tod je KV (Mittelwert und Median)

AAPV: Allgemeine ambulante Palliativversorgung, BQKPmV: Besonders qualifizierte und koordinierte palliativmedizinische Versorgung, KV: Kassenärztliche Vereinigung, PV: Palliativversorgung, SAPV: Spezialisierte ambulante Palliativversorgung, stat.PV: Stationäre Palliativversorgung im Krankenhaus

Quelle: eigene Abbildung

B.2 Qualitätsbezogene Outcomes

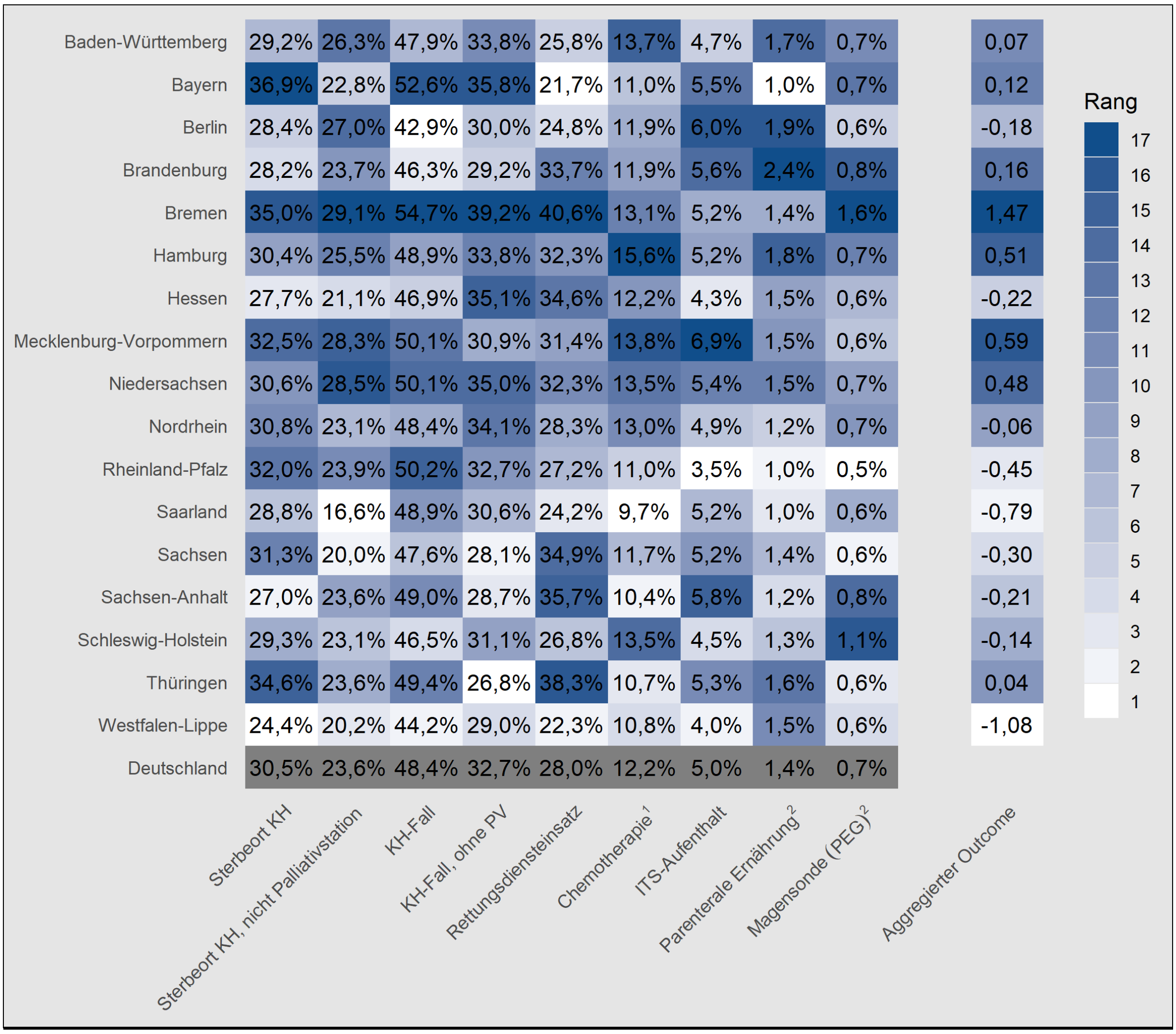

Abbildung B.2-1: Qualitätsbezogene Outcomeindikatoren je KV für Versicherte mit Palliativversorgung - ADJUSTIERT

Zeitlicher Bezug der Outcomeindikatoren: innerhalb von 30 Tagen vor dem Tod (Sterbeort: Todesdatum)
ITS: Intensivstation, KH: Krankenhaus, KV: Kassenärztliche Vereinigung, PEG: Perkutan-endoskopische Gastrostomie, PV: Palliativversorgung
[1]Versicherte mit Tumorerkrankung (ICD-10-GM: C00-C97), [2]Versicherte ohne gastrointestinalen Tumor oder gastrointestinale Metastasen (ICD-10-GM: C15-C26, C78.4-C78.8)
Quelle: eigene Abbildung

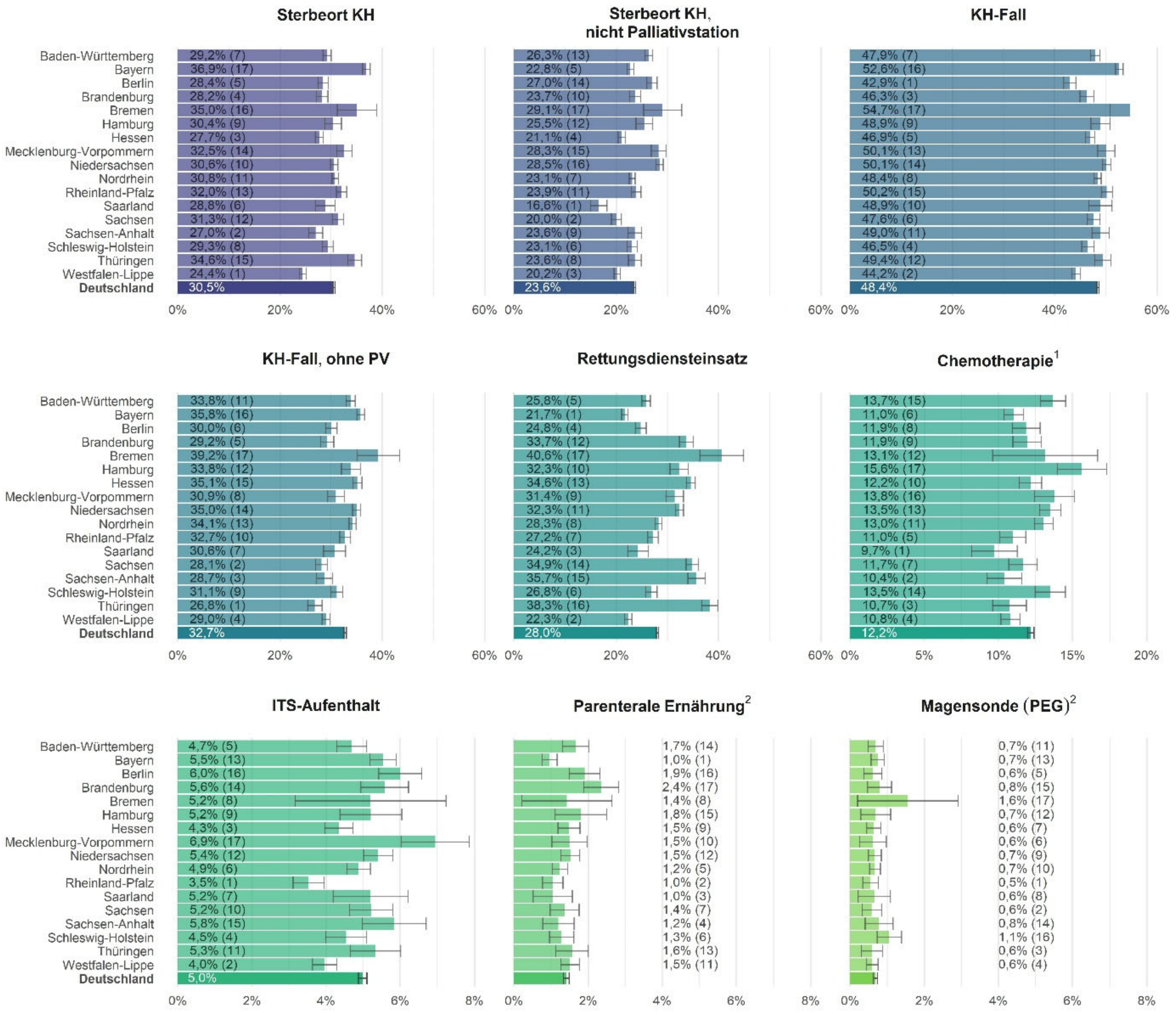

Abbildung B.2-2: Qualitätsbezogene Outcomeindikatoren je KV für Versicherte mit Palliativversorgung (mit 95%-Konfidenzintervall) - ADJUSTIERT

Zeitlicher Bezug der Outcomeindikatoren: innerhalb von 30 Tagen vor dem Tod (Sterbeort: Todesdatum)

ITS: Intensivstation, KH: Krankenhaus, KV: Kassenärztliche Vereinigung, PEG: Perkutan-endoskopische Gastrostomie, PV: Palliativversorgung

[1]Versicherte mit Tumorerkrankung (ICD-10-GM: C00-C97), [2]Versicherte ohne gastrointestinalen Tumor oder gastrointestinale Metastasen (ICD-10-GM: C15-C26, C78.4-C78.8)

Quelle: eigene Abbildung

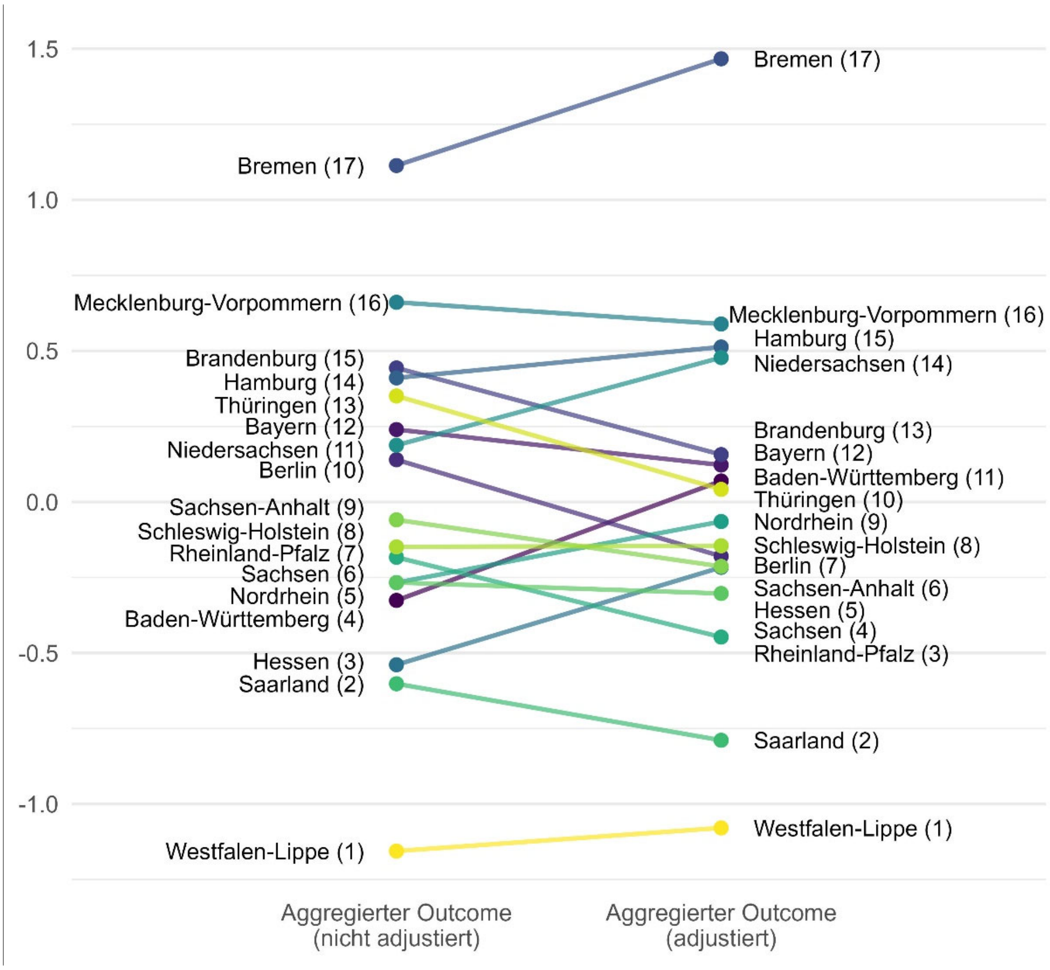

Abbildung B.2-3: Veränderung des aggregierten Outcomes nach Adjustierung

Skala: Je geringer die Werte auf dem aggregierten Outcome, desto höher die Versorgungsqualität. Negative Werte auf dem aggregierten Outcome legen nahe, dass die jeweilige KV besser als der Bundesschnitt liegt. Ein Abfall des aggregierten Outcomes nach Adjustierung legt nahe, dass die Versorgungsqualität besser ausfällt, wenn man um Patienten- und Wohnkreismerkmale adjustiert. Der Rang jeder KV wird in Klammern ausgewiesen.

Quelle: eigene Abbildung

B.3 Gesamtversorgungskosten der letzten drei Lebensmonate

Tabelle B.3-1: Gesamtversorgungskosten sowie Pflegekosten der drei letzten Lebensmonate je KV je Versichertem mit Palliativversorgung (Euro, MW, S)

KV	Ambulante ärztliche Versorgung		HKP		Krankenhaus		Rettungsdienst		Kranken-transport		Hospiz		Arzneimittel		Heilmittel		Hilfsmittel		Gesamt-versorgungs-kosten		Pflegeleistungen[1]	
	MW (S)	Rang	MW (S)	Rang	MW (S)	Rang	MW (S)	Rang	MW (S)	Rang	MW (S)	Rang	MW (S)	Rang	MW (S)	Rang	MW (S)	Rang	MW (S)	Rang	MW (S)	Rang
Baden-Württemberg	982 (1.381)	10	1.096 (3.502)	7	7.942 (10.891)	3	348 (650)	7	293 (655)	7	465 (2.683)	10	2.478 (5.167)	7	138 (331)	9	707 (1.445)	8	14.451 (14.029)	4	3.708 (2.508)	14
Bayern	1.068 (1.349)	12	1.069 (3.333)	5	8.863 (12.624)	12	343 (570)	4	230 (518)	2	202 (1.736)	1	1.953 (4.360)	1	120 (319)	6	595 (1.092)	2	14.444 (14.847)	3	3.434 (2.574)	10
Berlin	1.272 (1.351)	15	904 (3.668)	3	10.115 (16.675)	17	231 (376)	1	291 (576)	6	1.013 (3.964)	17	2.820 (5.365)	13	269 (591)	17	690 (1.318)	7	17.605 (18.593)	16	3.261 (2.476)	4
Brandenburg	756 (1.240)	1	2.057 (4.150)	17	9.402 (11.953)	15	542 (771)	15	319 (631)	12	642 (2.918)	15	3.229 (5.944)	17	119 (328)	5	886 (1.402)	16	17.951 (14.926)	17	3.252 (2.407)	3
Bremen	813 (1.102)	3	1.200 (2.932)	10	7.543 (8.429)	1	375 (525)	11	246 (500)	3	390 (2.408)	6	2.145 (4.766)	2	117 (256)	3	516 (782)	1	13.346 (11.102)	1	3.474 (2.393)	12
Hamburg	1.186 (1.524)	14	1.880 (3.971)	16	8.790 (12.479)	11	361 (488)	8	366 (731)	16	867 (3.518)	16	2.897 (5.159)	14	184 (428)	16	717 (1.308)	12	17.247 (15.142)	15	3.366 (2.549)	9
Hessen	807 (1.186)	2	1.855 (3.902)	15	8.134 (11.386)	4	552 (773)	17	230 (582)	1	524 (2.759)	11	2.570 (5.764)	10	103 (278)	1	714 (1.385)	11	15.487 (14.696)	8	3.471 (2.422)	11
Mecklenburg-Vorpommern	939 (1.323)	8	1.319 (3.917)	12	9.208 (14.071)	14	547 (817)	16	362 (785)	15	425 (2.062)	7	2.940 (5.042)	15	155 (360)	15	937 (1.717)	17	16.832 (16.227)	14	3.324 (2.369)	6
Niedersachsen	1.070 (1.479)	13	1.168 (3.238)	9	7.594 (10.863)	2	422 (673)	12	379 (733)	17	387 (2.294)	5	2.546 (4.963)	8	141 (366)	11	652 (1.260)	6	14.358 (13.737)	2	3.722 (2.419)	15
Nordrhein	2.401 (4.115)	17	538 (3.097)	1	8.539 (12.450)	9	361 (586)	9	308 (608)	10	535 (2.647)	13	2.470 (4.817)	6	139 (343)	10	637 (1.149)	4	15.928 (15.182)	13	3.814 (2.494)	17
Rheinland-Pfalz	945 (1.332)	9	994 (3.449)	4	8.527 (11.008)	8	336 (533)	3	284 (607)	5	429 (2.484)	8	2.395 (5.078)	5	131 (315)	7	616 (1.083)	3	14.656 (13.788)	6	3.334 (2.469)	7
Saarland	997 (1.565)	11	1.304 (3.174)	11	8.993 (12.663)	13	306 (498)	2	312 (768)	11	553 (2.642)	14	2.559 (5.812)	9	138 (325)	8	645 (1.311)	5	15.807 (15.781)	10	3.178 (2.370)	2
Sachsen	849 (1.207)	5	1.073 (3.299)	6	8.787 (12.038)	10	344 (498)	6	324 (596)	14	301 (1.943)	2	2.629 (5.389)	11	145 (333)	14	800 (1.376)	14	15.254 (14.566)	7	3.356 (2.422)	8
Sachsen-Anhalt	893 (1.367)	6	1.352 (3.970)	13	8.308 (10.417)	5	464 (673)	14	321 (639)	13	452 (2.387)	9	2.941 (5.213)	16	143 (335)	13	721 (1.290)	13	15.595 (13.315)	9	3.476 (2.455)	13
Schleswig-Holstein	901 (1.259)	7	1.774 (4.032)	14	8.488 (11.896)	7	456 (735)	13	263 (563)	4	360 (2.161)	4	2.806 (5.540)	12	141 (342)	12	708 (1.241)	9	15.897 (14.644)	12	2.957 (2.404)	1
Thüringen	842 (1.496)	4	1.164 (3.847)	8	9.618 (12.149)	16	343 (464)	5	306 (651)	8	337 (1.928)	3	2.248 (4.213)	3	113 (281)	2	845 (1.556)	15	15.817 (14.379)	11	3.283 (2.400)	5
Westfalen-Lippe	1.448 (1.414)	16	550 (3.119)	2	8.309 (11.437)	6	368 (654)	10	307 (597)	9	534 (2.678)	12	2.257 (4.328)	4	118 (294)	4	709 (1.139)	10	14.599 (13.795)	5	3.751 (2.558)	16

KV	Ambulante ärztliche Versorgung		HKP		Krankenhaus		Rettungsdienst		Kranken-transport		Hospiz		Arzneimittel		Heilmittel		Hilfsmittel		Gesamt-versorgungs-kosten		Pflegeleistungen[1]	
	MW (S)	Rang	MW (S)	Rang	MW (S)	Rang	MW (S)	Rang	MW (S)	Rang	MW (S)	Rang	MW (S)	Rang	MW (S)	Rang	MW (S)	Rang	MW (S)	Rang	MW (S)	Rang
**Deutschland**	**1.237 (2.078)**	-	**1.103 (3.502)**	-	**8.547 (12.112)**	-	**388 (631)**	-	**294 (618)**	-	**459 (2.528)**	-	**2.465 (4.999)**	-	**137 (346)**	-	**687 (1.263)**	-	**15.317 (14.757)**	-	**3.522 (2.493)**	-

HKP: Häusliche Krankenpflege (SGB V), KV: Kassenärztliche Vereinigung, MW: Mittelwert, S: Standardabweichung, SGB XI: Sozialgesetzbuch XI
[1]Kosten der Pflegeleistungen nur für die Subpopulation der Verstorbenen aus 2018 und 2019 verfügbar; Grundgesamtheit reduziert sich hier auf Verstorbene aus 2018 und 2019.

Tabelle B.3-2: Gesamtversorgungskosten sowie Pflegekosten der letzten drei Lebensmonate je KV je Versichertem mit Palliativversorgung (Euro, Median, IQA)

KV	Ambulante ärztliche Versorgung		HKP		Krankenhaus		Rettungsdienst		Krankentransport		Hospiz		Arzneimittel		Heilmittel		Hilfsmittel		Gesamt-versorgungs-kosten		Pflegeleistungen[1]	
	Median (IQA)	Rang	Median (IQA)	Rang	Median (IQA)	Rang	Median (IQA)	Rang	Median (IQA)	Rang	Median (IQA)	Rang	Median (IQA)	Rang	Median (IQA)	Rang	Median (IQA)	Rang	Median (IQA)	Rang	Median (IQA)	Rang
Baden-Württemberg	651 (620)	11	19 (1.483)	3	4.790 (10.985)	2	0 (478)	1	117 (317)	8	0 (0)	1	718 (1.527)	3	0 (127)	1	238 (763)	6	10.542 (15.399)	2	3.543 (4.406)	14
Bayern	756 (712)	14	0 (907)	1	5.402 (11.321)	7	0 (745)	1	92 (240)	2	0 (0)	1	594 (1.067)	2	0 (91)	1	200 (671)	2	10.393 (14.887)	1	3.233 (4.798)	11
Berlin	953 (1.028)	15	0 (614)	1	6.189 (12.372)	15	0 (319)	1	131 (314)	10	0 (0)	1	813 (2.063)	12	0 (297)	1	288 (787)	9	13.427 (16.803)	15	2.978 (4.352)	5
Brandenburg	454 (502)	1	1.019 (2.666)	12	6.464 (11.631)	16	181 (866)	3	103 (349)	5	0 (0)	1	879 (2.436)	13	0 (85)	1	405 (1.026)	17	14.493 (16.497)	17	2.888 (3.973)	3
Bremen	540 (535)	3	83 (1.037)	4	5.044 (9.717)	4	297 (594)	6	100 (285)	4	0 (0)	1	585 (978)	1	0 (111)	1	199 (614)	1	10.847 (14.692)	4	3.117 (4.024)	10
Hamburg	754 (887)	13	205 (1.912)	7	5.768 (10.341)	14	0 (463)	1	177 (417)	17	0 (0)	1	880 (2.131)	14	0 (180)	1	264 (786)	7	13.644 (16.418)	16	3.023 (4.642)	7
Hessen	543 (524)	4	791 (2.243)	11	4.842 (10.810)	3	406 (800)	7	76 (210)	1	0 (0)	1	756 (1.540)	8	0 (62)	1	294 (788)	11	11.925 (15.412)	8	3.236 (4.168)	12
Mecklenburg-Vorpommern	612 (621)	8	117 (1.269)	6	5.710 (10.387)	12	0 (930)	1	148 (362)	14	0 (0)	1	988 (2.369)	17	0 (157)	1	357 (1.029)	16	13.081 (15.038)	14	2.995 (3.879)	6
Niedersachsen	696 (687)	12	15 (1.216)	2	4.722 (9.965)	1	0 (622)	1	167 (431)	16	0 (0)	1	731 (1.582)	7	0 (124)	1	224 (712)	4	10.835 (14.260)	3	3.611 (4.295)	16
Nordrhein	974 (1.645)	16	0 (125)	1	5.392 (10.989)	6	0 (567)	1	138 (359)	13	0 (0)	1	775 (1.619)	9	0 (115)	1	237 (723)	5	12.388 (16.168)	11	3.689 (4.346)	17
Rheinland-Pfalz	622 (607)	10	0 (724)	1	5.642 (11.125)	11	0 (587)	1	111 (301)	6	0 (0)	1	718 (1.415)	4	0 (113)	1	219 (698)	3	11.394 (15.427)	6	3.080 (4.234)	8

KV	Ambulante ärztliche Versorgung		HKP		Krankenhaus		Rettungsdienst		Krankentransport		Hospiz		Arzneimittel		Heilmittel		Hilfsmittel		Gesamt-versorgungs-kosten		Pflegeleistungen[1]	
	Median (IQA)	Rang	Median (IQA)	Rang	Median (IQA)	Rang	Median (IQA)	Rang	Median (IQA)	Rang	Median (IQA)	Rang	Median (IQA)	Rang	Median (IQA)	Rang	Median (IQA)	Rang	Median (IQA)	Rang	Median (IQA)	Rang
Saarland	616 (616)	9	0 (1.375)	1	5.737 (10.061)	13	0 (441)	1	115 (317)	7	0 (0)	1	883 (1.685)	15	0 (112)	1	311 (802)	15	12.077 (14.876)	9	2.847 (3.870)	2
Sachsen	562 (558)	5	318 (1.586)	9	5.604 (10.579)	10	156 (515)	2	153 (370)	15	0 (0)	1	777 (1.725)	10	0 (141)	1	289 (849)	10	11.545 (15.062)	7	3.094 (4.237)	9
Sachsen-Anhalt	567 (567)	6	424 (1.462)	10	5.475 (9.619)	9	215 (684)	4	126 (351)	9	0 (0)	1	893 (2.170)	16	0 (132)	1	298 (776)	12	12.430 (14.991)	12	3.309 (4.133)	13
Schleswig-Holstein	602 (576)	7	103 (2.065)	5	5.213 (10.951)	5	0 (743)	1	99 (278)	3	0 (0)	1	778 (1.859)	11	0 (129)	1	309 (795)	14	12.515 (16.102)	13	2.631 (4.079)	1
Thüringen	531 (486)	2	229 (1.730)	8	6.482 (11.028)	17	227 (552)	5	133 (327)	11	0 (0)	1	727 (1.479)	5	0 (92)	1	280 (942)	8	12.306 (15.786)	10	2.977 (4.029)	4
Westfalen-Lippe	1.154 (871)	17	0 (318)	1	5.412 (11.222)	8	0 (558)	1	133 (362)	12	0 (0)	1	730 (1.352)	6	0 (92)	1	304 (800)	13	11.116 (14.627)	5	3.608 (4.466)	15
**Deutschland**	**725 (812)**	-	**0 (1.196)**	-	**5.366 (10.945)**	-	**0 (635)**	-	**118 (322)**	-	**0 (0)**	-	**735 (1.545)**	-	**0 (114)**	-	**258 (768)**	-	**11.654 (15.474)**	-	**3.312 (4.435)**	-

HKP: Häusliche Krankenpflege (SGB V), IQA: Interquartilsabstand, KV: Kassenärztliche Vereinigung, SGB XI: Sozialgesetzbuch XI

[1]Kosten der Pflegeleistungen nur für die Subpopulation der Verstorbenen aus 2018 und 2019 verfügbar; Grundgesamtheit reduziert sich hier auf Verstorbene aus 2018 und 2019.

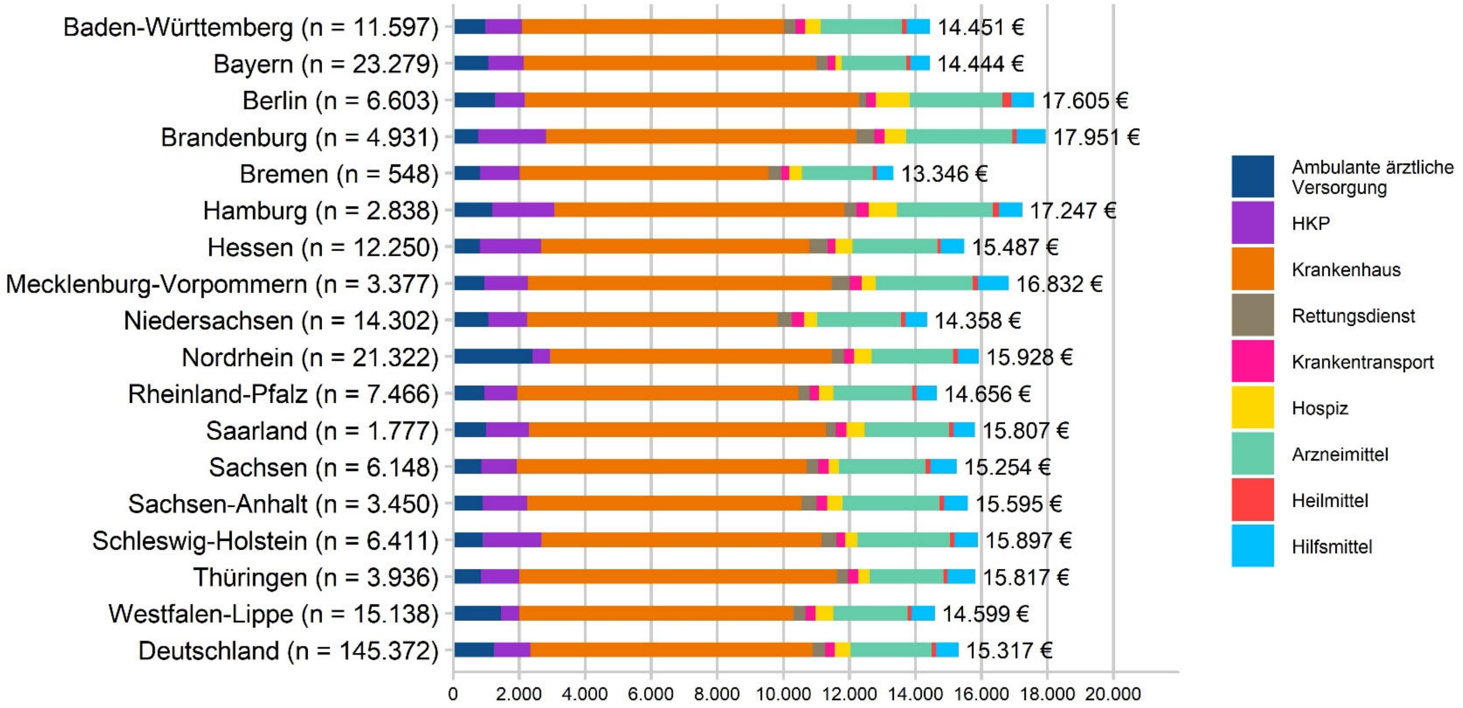

Abbildung B.3-1: Gesamtversorgungskosten der letzten drei Lebensmonate nach Leistungsbereichen je KV je Versichertem mit Palliativversorgung

Berücksichtigte Leistungssektoren: Ambulante ärztliche Versorgung, HKP (Häusliche Krankenpflege, SGB V), Krankenhaus, Rettungsdienst, Krankentransport, Hospiz, Arzneimittel, Heilmittel, Hilfsmittel, exkl. SGB XI-Pflege. Darin enthalten sind auch die Palliativversorgungskosten der letzten drei Lebensmonate. KV: Kassenärztliche Vereinigung

Quelle: eigene Abbildung

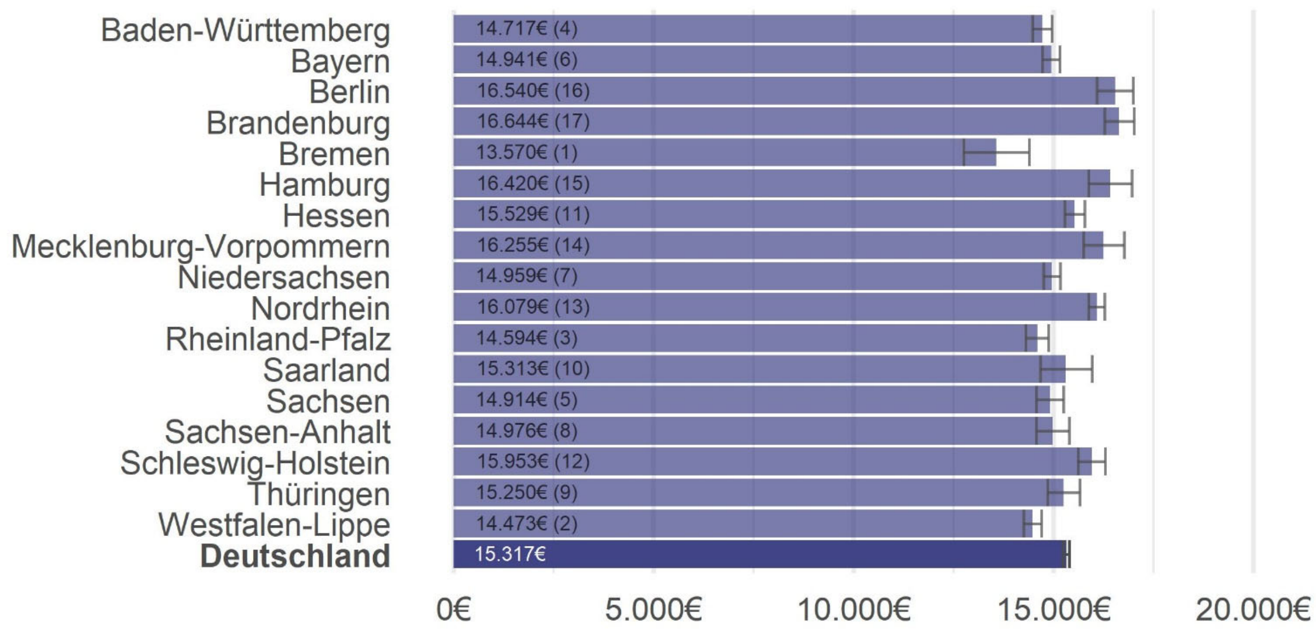

Abbildung B.3-2: Gesamtversorgungskosten der letzten drei Lebensmonate je KV je Versichertem mit Palliativversorgung (Mittelwert (Rang), 95%-Konfidenzintervall) - ADJUSTIERT

KV: Kassenärztliche Vereinigung

Quelle: eigene Abbildung

B.4 Palliativversorgungskosten des letzten Lebensjahres

Palliativversorgungskosten je Versichertem mit Palliativversorgung

Tabelle B.4-1: Palliativversorgungskosten des letzten Lebensjahres je KV je Versichertem mit Palliativversorgung (Euro, MW, S)

	AAPV		BQKPmV		SAPV		HKPpall		HKP_aP		stat.PV		Hospiz		PV¹		HKP gesamt	
**KV**	**MW (S)**	**Rang**	**MW (S)**	**Rang**	**MW (S)**	**Rang**	**MW (S)**	**Rang**	**MW (S)**	**Rang**	**MW (S)**	**Rang**	**MW (S)**	**Rang**	**MW (S)**	**Rang**	**MW (S)**	**Rang**
Baden-Württemberg	78 (113)	10	19 (74)	11	772 (1.513)	3	0 (0)	1	167 (1.994)	9	660 (2.140)	14	568 (3.834)	9	2.263 (5.371)	7	1.446 (11.629)	10
Bayern	113 (154)	15	9 (65)	5	842 (1.955)	5	0 (67)	6	156 (1.817)	8	764 (2.202)	16	256 (2.653)	1	2.141 (4.653)	4	1.245 (10.599)	7
Berlin	77 (139)	9	16 (70)	10	1.061 (2.493)	10	0 (15)	5	200 (2.005)	15	559 (1.255)	9	1.307 (6.419)	17	3.219 (7.769)	13	1.781 (12.039)	16
Brandenburg	45 (88)	2	13 (56)	6	2.516 (4.485)	17	0 (10)	3	190 (2.109)	13	599 (1.415)	10	866 (5.131)	15	4.228 (7.724)	16	1.640 (13.171)	13
Bremen	73 (105)	8	7 (40)	2	1.031 (2.985)	8	0 (0)	1	124 (1.130)	4	289 (825)	1	559 (4.784)	8	2.083 (6.152)	2	994 (3.654)	2
Hamburg	96 (162)	14	16 (68)	9	2.241 (6.009)	16	0 (0)	1	69 (673)	1	810 (2.051)	17	1.090 (5.068)	16	4.322 (8.639)	17	1.053 (8.087)	3
Hessen	63 (109)	5	21 (88)	14	1.877 (3.369)	14	0 (7)	2	186 (2.146)	11	460 (1.512)	5	657 (4.056)	12	3.265 (6.602)	14	1.285 (10.317)	8
Mecklenburg-Vorpommern	71 (106)	7	20 (88)	12	1.034 (2.684)	9	0 (0)	1	233 (1.793)	17	412 (937)	3	545 (3.349)	7	2.315 (4.977)	8	2.005 (13.155)	17
Niedersachsen	84 (122)	12	15 (73)	8	1.259 (3.483)	11	0 (33)	4	126 (1.588)	5	387 (1.316)	2	476 (3.397)	5	2.348 (5.620)	10	1.146 (9.754)	6
Nordrhein	339 (704)	17	8 (46)	3	1.813 (5.807)	13	111 (916)	9	168 (1.943)	10	659 (2.139)	13	682 (4.123)	13	3.780 (8.191)	15	1.427 (9.723)	9
Rheinland-Pfalz	88 (139)	13	22 (93)	15	868 (3.277)	6	1 (55)	7	137 (1.580)	6	723 (2.080)	15	493 (3.208)	6	2.331 (5.611)	9	1.110 (9.418)	4
Saarland	62 (112)	4	35 (132)	17	1.524 (3.804)	12	0 (0)	1	77 (1.679)	2	465 (963)	6	699 (4.048)	14	2.862 (6.299)	11	528 (5.442)	1
Sachsen	65 (99)	6	21 (93)	13	673 (922)	2	0 (0)	1	188 (1.658)	12	495 (1.077)	7	374 (2.923)	2	1.816 (3.841)	1	1.643 (10.885)	14
Sachsen-Anhalt	44 (68)	1	23 (79)	16	965 (2.626)	7	2 (92)	8	191 (1.508)	14	433 (909)	4	581 (3.788)	10	2.237 (5.043)	5	1.566 (9.687)	11
Schleswig-Holstein	79 (137)	11	8 (52)	4	1.915 (4.379)	15	0 (0)	1	120 (1.735)	3	623 (1.749)	11	447 (3.282)	4	3.192 (6.577)	12	1.120 (10.174)	5
Thüringen	61 (88)	3	13 (66)	7	809 (1.075)	4	0 (0)	1	223 (3.510)	16	645 (1.486)	12	386 (2.511)	3	2.137 (4.907)	3	1.644 (12.665)	15
Westfalen-Lippe	145 (224)	16	2 (35)	1	572 (596)	1	188 (1.081)	10	153 (1.713)	7	558 (1.732)	8	635 (3.633)	11	2.253 (4.831)	6	1.608 (10.853)	12
**Deutschland**	**126 (314)**	**-**	**13 (69)**	**-**	**1.230 (3.449)**	**-**	**36 (500)**	**-**	**162 (1.888)**	**-**	**598 (1.801)**	**-**	**573 (3.799)**	**-**	**2.737 (6.177)**	**-**	**1.384 (10.591)**	**-**

AAPV: Allgemeine ambulante Palliativversorgung, BQKPmV: Besonders qualifizierte und koordinierte palliativmedizinische Versorgung, HKP: Häusliche Krankenpflege (SGB V), HKP gesamt: sämtliche HKP-Leistungen des letzten Lebensjahres (nachrichtlich), HKPpall: palliative HKP-Leistungen, HKP_aP: HKP-Leistungen ab erster ambulanter Palliativleistung, KV: Kassenärztliche Vereinigung, MW: Mittelwert, PV: Palliativversorgung, S: Standardabweichung, SAPV: Spezialisierte ambulante Palliativversorgung, stat.PV: stationäre Palliativversorgung im Krankenhaus

[1] *als Summe aus AAPV, BQKPmV, SAPV, HKPpall, HKP_aP, stat.PV und Hospiz*

Tabelle B.4-2: Palliativversorgungskosten des letzten Lebensjahres je KV je Versichertem mit Palliativversorgung (Euro, Median, IQA)

	AAPV		BQKPmV		SAPV		HKPpall		HKP_aP		stat.PV		Hospiz		PV[1]		HKP gesamt	
KV	Median (IQA)	Rang	Median (IQA)	Rang	Median (IQA)	Rang	Median (IQA)	Rang	Median (IQA)	Rang	Median (IQA)	Rang	Median (IQA)	Rang	Median (IQA)	Rang	Median (IQA)	Rang
Baden-Württemberg	50 (93)	8	0 (0)	1	0 (1.430)	1	0 (0)	1	0 (0)	1	0 (0)	1	0 (0)	1	593 (2.181)	6	0 (420)	1
Bayern	66 (104)	12	0 (0)	1	0 (27)	1	0 (0)	1	0 (0)	1	0 (0)	1	0 (0)	1	321 (2.710)	3	0 (141)	1
Berlin	36 (87)	3	0 (0)	1	180 (1.039)	2	0 (0)	1	0 (0)	1	0 (600)	1	0 (0)	1	902 (2.723)	10	0 (121)	1
Brandenburg	0 (53)	1	0 (0)	1	937 (2.769)	5	0 (0)	1	0 (0)	1	0 (800)	1	0 (0)	1	1.553 (4.397)	16	0 (358)	1
Bremen	50 (85)	9	0 (0)	1	0 (388)	1	0 (0)	1	0 (0)	1	0 (0)	1	0 (0)	1	208 (1.640)	1	0 (583)	1
Hamburg	49 (101)	5	0 (0)	1	0 (1.641)	1	0 (0)	1	0 (0)	1	0 (1.134)	1	0 (0)	1	1.153 (4.370)	13	0 (216)	1
Hessen	36 (76)	4	0 (0)	1	190 (2.028)	3	0 (0)	1	0 (0)	1	0 (0)	1	0 (0)	1	1.636 (3.247)	17	0 (290)	1
Mecklenburg-Vorpommern	50 (85)	6	0 (0)	1	0 (631)	1	0 (0)	1	0 (0)	1	0 (0)	1	0 (0)	1	479 (2.209)	5	0 (831)	1
Niedersachsen	53 (89)	11	0 (0)	1	0 (885)	1	0 (0)	1	0 (0)	1	0 (0)	1	0 (0)	1	344 (2.005)	4	0 (227)	1
Nordrhein	85 (363)	13	0 (0)	1	0 (776)	1	0 (0)	1	0 (0)	1	0 (0)	1	0 (0)	1	944 (3.106)	11	0 (396)	1
Rheinland-Pfalz	52 (101)	10	0 (0)	1	0 (0)	1	0 (0)	1	0 (0)	1	0 (0)	1	0 (0)	1	267 (1.907)	2	0 (165)	1
Saarland	49 (75)	5	0 (0)	1	0 (1.323)	1	0 (0)	1	0 (0)	1	0 (225)	1	0 (0)	1	729 (3.014)	7	0 (40)	1
Sachsen	49 (77)	5	0 (0)	1	0 (1.505)	1	0 (0)	1	0 (0)	1	0 (0)	1	0 (0)	1	1.358 (1.957)	15	0 (762)	1
Sachsen-Anhalt	26 (63)	2	0 (0)	1	0 (1.112)	1	0 (0)	1	0 (0)	1	0 (0)	1	0 (0)	1	955 (2.038)	12	0 (737)	1
Schleswig-Holstein	50 (89)	7	0 (0)	1	0 (1.972)	1	0 (0)	1	0 (0)	1	0 (0)	1	0 (0)	1	833 (3.360)	8	0 (171)	1
Thüringen	49 (78)	5	0 (0)	1	0 (1.786)	1	0 (0)	1	0 (0)	1	0 (1.134)	1	0 (0)	1	1.307 (2.470)	14	0 (370)	1
Westfalen-Lippe	90 (126)	14	0 (0)	1	525 (515)	4	0 (0)	1	0 (0)	1	0 (0)	1	0 (0)	1	865 (1.286)	9	0 (742)	1
**Deutschland**	**53 (120)**	-	**0 (0)**	-	**0 (1.125)**	-	**0 (0)**	-	**0 (0)**	-	**0 (0)**	-	**0 (0)**	-	**766 (2.533)**	-	**0 (343)**	-

AAPV: Allgemeine ambulante Palliativversorgung, BQKPmV: Besonders qualifizierte und koordinierte palliativmedizinische Versorgung, HKP: Häusliche Krankenpflege (SGB V), HKP gesamt: sämtliche HKP-Leistungen des letzten Lebensjahres (nachrichtlich), HKPpall: palliative HKP-Leistungen, HKP_aP: HKP-Leistungen ab erster ambulanter Palliativleistung, IQA: Interquartilsabstand, KV: Kassenärztliche Vereinigung, PV: Palliativversorgung, SAPV: Spezialisierte ambulante Palliativversorgung, stat.PV: stationäre Palliativversorgung im Krankenhaus

[1]als Summe aus AAPV, BQKPmV, SAPV, HKPpall, HKP_aP, stat.PV und Hospiz

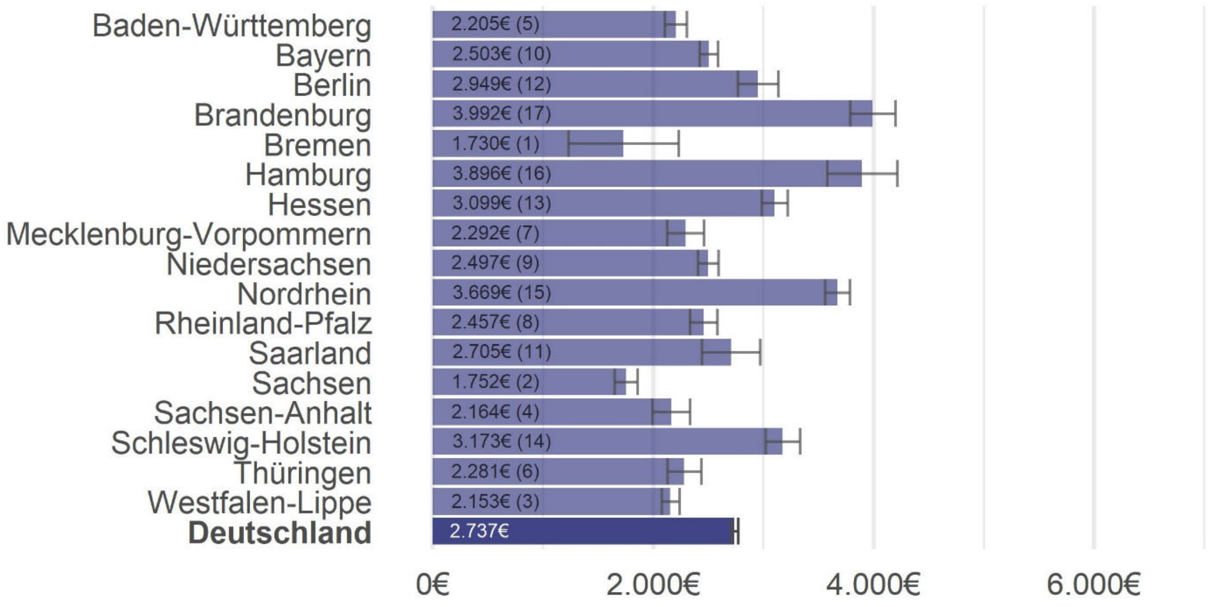

Abbildung B.4-1: Palliativversorgungskosten des letzten Lebensjahres je KV je Versichertem mit Palliativversorgung (Mittelwert (Rang), 95% Konfidenzintervall) - ADJUSTIERT

KV: Kassenärztliche Vereinigung

Quelle: eigene Abbildung

Palliativversorgungskosten je Versichertem mit der jeweiligen Palliativversorgungsform

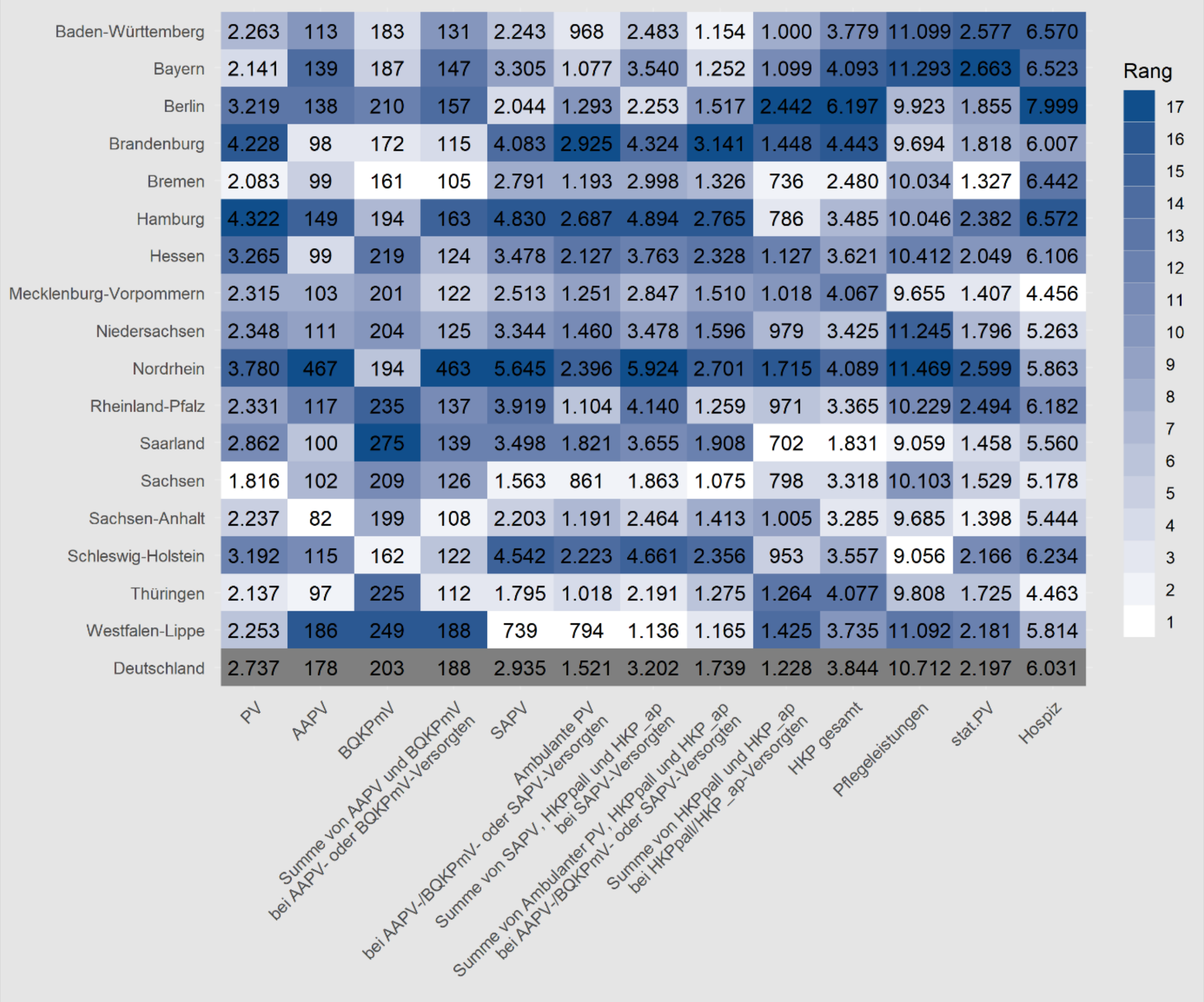

KV	PV	AAPV	BQKPmV	Summe von AAPV und BQKPmV bei AAPV- oder BQKPmV-Versorgten	SAPV	Ambulante PV bei AAPV-/BQKPmV- oder SAPV-Versorgten	Summe von SAPV, HKPpall und HKP _ap bei SAPV-Versorgten	Summe von Ambulanter PV, HKPpall und HKP _ap bei AAPV-/BQKPmV- oder SAPV-Versorgten	Summe von HKPpall und HKP _ap bei HKPpall/HKP _ap-Versorgten	HKP gesamt	Pflegeleistungen	stat.PV	Hospiz
Baden-Württemberg	2.263	113	183	131	2.243	968	2.483	1.154	1.000	3.779	11.099	2.577	6.570
Bayern	2.141	139	187	147	3.305	1.077	3.540	1.252	1.099	4.093	11.293	2.663	6.523
Berlin	3.219	138	210	157	2.044	1.293	2.253	1.517	2.442	6.197	9.923	1.855	7.999
Brandenburg	4.228	98	172	115	4.083	2.925	4.324	3.141	1.448	4.443	9.694	1.818	6.007
Bremen	2.083	99	161	105	2.791	1.193	2.998	1.326	736	2.480	10.034	1.327	6.442
Hamburg	4.322	149	194	163	4.830	2.687	4.894	2.765	786	3.485	10.046	2.382	6.572
Hessen	3.265	99	219	124	3.478	2.127	3.763	2.328	1.127	3.621	10.412	2.049	6.106
Mecklenburg-Vorpommern	2.315	103	201	122	2.513	1.251	2.847	1.510	1.018	4.067	9.655	1.407	4.456
Niedersachsen	2.348	111	204	125	3.344	1.460	3.478	1.596	979	3.425	11.245	1.796	5.263
Nordrhein	3.780	467	194	463	5.645	2.396	5.924	2.701	1.715	4.089	11.469	2.599	5.863
Rheinland-Pfalz	2.331	117	235	137	3.919	1.104	4.140	1.259	971	3.365	10.229	2.494	6.182
Saarland	2.862	100	275	139	3.498	1.821	3.655	1.908	702	1.831	9.059	1.458	5.560
Sachsen	1.816	102	209	126	1.563	861	1.863	1.075	798	3.318	10.103	1.529	5.178
Sachsen-Anhalt	2.237	82	199	108	2.203	1.191	2.464	1.413	1.005	3.285	9.685	1.398	5.444
Schleswig-Holstein	3.192	115	162	122	4.542	2.223	4.661	2.356	953	3.557	9.056	2.166	6.234
Thüringen	2.137	97	225	112	1.795	1.018	2.191	1.275	1.264	4.077	9.808	1.725	4.463
Westfalen-Lippe	2.253	186	249	188	739	794	1.136	1.165	1.425	3.735	11.092	2.181	5.814
Deutschland	2.737	178	203	188	2.935	1.521	3.202	1.739	1.228	3.844	10.712	2.197	6.031

Abbildung B.4-2: Mittelwerte ausgewählter Bestandteile der Palliativversorgungskosten im letzten Lebensjahr je KV je Versichertem mit der jeweiligen Versorgungsform (Euro)

AAPV: Allgemeine ambulante Palliativversorgung, BQKPmV: Besonders qualifizierte und koordinierte palliativmedizinische Versorgung, HKP: Häusliche Krankenpflege (SGB V), HKP gesamt: sämtliche HKP-Leistungen des letzten Lebensjahres (nachrichtlich), HKPpall: palliative HKP-Leistungen, HKP_aP: HKP-Leistungen ab erster ambulanter Palliativleistung, KV: Kassenärztliche Vereinigung, PV: Palliativversorgung, SAPV: Spezialisierte ambulante Palliativversorgung, stat.PV: stationäre Palliativversorgung im Krankenhaus

Quelle: eigene Abbildung

	PV	AAPV	BQKPmV	Summe von AAPV und BQKPmV bei AAPV- oder BQKPmV-Versorgten	SAPV	Ambulante PV bei AAPV-/BQKPmV- oder SAPV-Versorgten	Summe von SAPV, HKPpall und HKP_ap bei SAPV-Versorgten	Summe von Ambulanter PV, HKPpall und HKP_ap bei AAPV-/BQKPmV- oder SAPV-Versorgten	Summe von HKPpall und HKP_ap bei HKPpall/HKP_ap-Versorgten	HKP gesamt	Pflegeleistungen	stat.PV	Hospiz
Baden-Württemberg	2.205	116	176	133	2.283	969	2.538	1.174	1.048	3.775	10.923	2.435	6.292
Bayern	2.503	145	185	154	3.387	1.231	3.633	1.450	1.184	4.065	10.276	2.787	6.239
Berlin	2.949	137	209	156	1.954	1.186	2.178	1.408	2.334	6.145	10.541	1.718	8.140
Brandenburg	3.992	91	179	108	3.955	2.737	4.183	2.933	1.578	4.861	10.916	1.932	6.359
Bremen	1.730	97	161	102	2.594	1.069	2.791	1.180	757	2.291	11.061	1.114	5.639
Hamburg	3.896	148	179	161	4.732	2.469	4.816	2.564	952	3.587	10.660	2.274	6.049
Hessen	3.099	99	215	124	3.543	2.055	3.833	2.254	1.102	3.398	10.794	1.943	5.811
Mecklenburg-Vorpommern	2.292	96	211	115	2.288	1.190	2.580	1.415	1.120	4.472	10.512	1.552	4.831
Niedersachsen	2.497	110	205	124	3.214	1.515	3.331	1.656	1.057	3.578	10.722	1.817	5.249
Nordrhein	3.669	466	192	462	5.630	2.383	5.902	2.674	1.596	3.852	11.349	2.483	5.812
Rheinland-Pfalz	2.457	117	239	138	3.861	1.169	4.054	1.306	953	3.208	10.305	2.560	6.302
Saarland	2.705	100	276	138	3.445	1.758	3.576	1.806	682	1.496	10.020	1.450	5.741
Sachsen	1.752	99	209	123	1.467	804	1.767	1.013	893	3.778	10.783	1.572	5.351
Sachsen-Anhalt	2.164	75	215	102	2.040	1.119	2.268	1.303	989	3.668	10.597	1.548	6.078
Schleswig-Holstein	3.173	112	169	120	4.352	2.188	4.443	2.311	1.007	3.594	9.398	2.250	6.196
Thüringen	2.281	94	231	109	1.760	1.037	2.137	1.279	1.255	4.278	10.414	1.875	4.932
Westfalen-Lippe	2.153	183	247	185	920	770	1.337	1.122	1.330	3.575	11.155	2.119	5.959
Deutschland	2.737	178	203	188	2.935	1.521	3.202	1.739	1.228	3.844	10.712	2.197	6.031

Rang: 17 16 15 14 13 12 11 10 9 8 7 6 5 4 3 2 1

Abbildung B.4-3: Mittelwerte ausgewählter Bestandteile der Palliativversorgungskosten im letzten Lebensjahr je KV je Versichertem mit der jeweiligen Versorgungsform (Euro) - ADJUSTIERT

AAPV: Allgemeine ambulante Palliativversorgung, BQKPmV: Besonders qualifizierte und koordinierte palliativmedizinische Versorgung, HKP: Häusliche Krankenpflege (SGB V), HKP gesamt: sämtliche HKP-Leistungen des letzten Lebensjahres (nachrichtlich), HKPpall: palliative HKP-Leistungen, HKP_aP: HKP-Leistungen ab erster ambulanter Palliativleistung, KV: Kassenärztliche Vereinigung, PV: Palliativversorgung, SAPV: Spezialisierte ambulante Palliativversorgung, stat.PV: stationäre Palliativversorgung im Krankenhaus

Quelle: eigene Abbildung

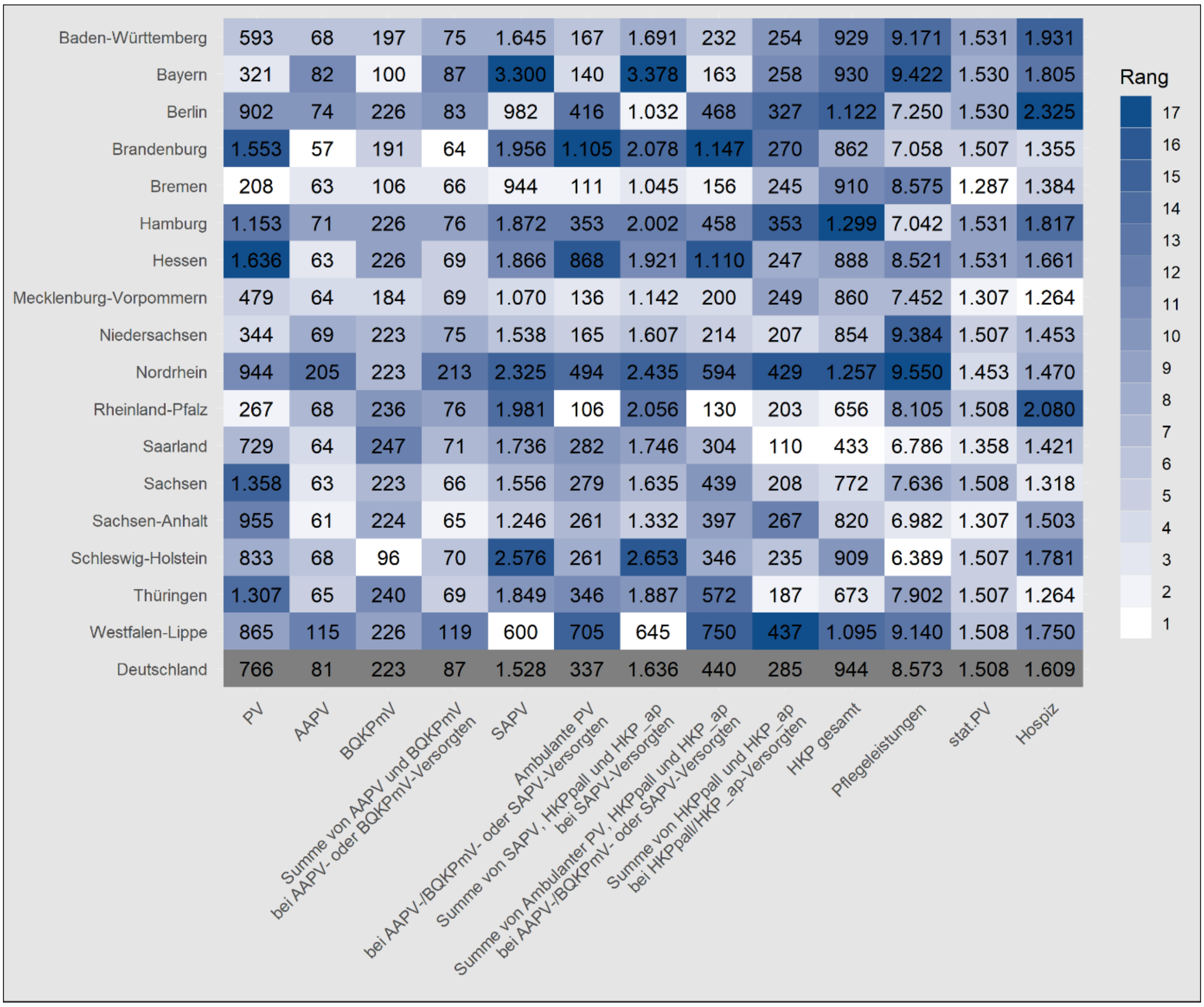

	PV	AAPV	BQKPmV	Summe von AAPV und BQKPmV bei AAPV- oder BQKPmV-Versorgten	SAPV	Ambulante PV bei AAPV-/BQKPmV- oder SAPV-Versorgten	Summe von SAPV, HKPpall und HKP_ap bei SAPV-Versorgten	Summe von Ambulanter PV, HKPpall und HKP_ap bei AAPV-/BQKPmV- oder SAPV-Versorgten	Summe von HKPpall und HKP_ap bei HKPpall/HKP_ap-Versorgten	HKP gesamt	Pflegeleistungen	stat.PV	Hospiz
Baden-Württemberg	593	68	197	75	1.645	167	1.691	232	254	929	9.171	1.531	1.931
Bayern	321	82	100	87	3.300	140	3.378	163	258	930	9.422	1.530	1.805
Berlin	902	74	226	83	982	416	1.032	468	327	1.122	7.250	1.530	2.325
Brandenburg	1.553	57	191	64	1.956	1.105	2.078	1.147	270	862	7.058	1.507	1.355
Bremen	208	63	106	66	944	111	1.045	156	245	910	8.575	1.287	1.384
Hamburg	1.153	71	226	76	1.872	353	2.002	458	353	1.299	7.042	1.531	1.817
Hessen	1.636	63	226	69	1.866	868	1.921	1.110	247	888	8.521	1.531	1.661
Mecklenburg-Vorpommern	479	64	184	69	1.070	136	1.142	200	249	860	7.452	1.307	1.264
Niedersachsen	344	69	223	75	1.538	165	1.607	214	207	854	9.384	1.507	1.453
Nordrhein	944	205	223	213	2.325	494	2.435	594	429	1.257	9.550	1.453	1.470
Rheinland-Pfalz	267	68	236	76	1.981	106	2.056	130	203	656	8.105	1.508	2.080
Saarland	729	64	247	71	1.736	282	1.746	304	110	433	6.786	1.358	1.421
Sachsen	1.358	63	223	66	1.556	279	1.635	439	208	772	7.636	1.508	1.318
Sachsen-Anhalt	955	61	224	65	1.246	261	1.332	397	267	820	6.982	1.307	1.503
Schleswig-Holstein	833	68	96	70	2.576	261	2.653	346	235	909	6.389	1.507	1.781
Thüringen	1.307	65	240	69	1.849	346	1.887	572	187	673	7.902	1.507	1.264
Westfalen-Lippe	865	115	226	119	600	705	645	750	437	1.095	9.140	1.508	1.750
Deutschland	766	81	223	87	1.528	337	1.636	440	285	944	8.573	1.508	1.609

Abbildung B.4-4: Mediane ausgewählter Bestandteile der Palliativversorgungskosten im letzten Lebensjahr je KV je Versichertem mit der jeweiligen Versorgungsform (Euro)

AAPV: Allgemeine ambulante Palliativversorgung, BQKPmV: Besonders qualifizierte und koordinierte palliativmedizinische Versorgung, HKP: Häusliche Krankenpflege (SGB V), HKP gesamt: sämtliche HKP-Leistungen des letzten Lebensjahres (nachrichtlich), HKPpall: palliative HKP-Leistungen, HKP_aP: HKP-Leistungen ab erster ambulanter Palliativleistung, KV: Kassenärztliche Vereinigung, PV: Palliativversorgung, SAPV: Spezialisierte ambulante Palliativversorgung, stat.PV: stationäre Palliativversorgung im Krankenhaus
Quelle: eigene Abbildung

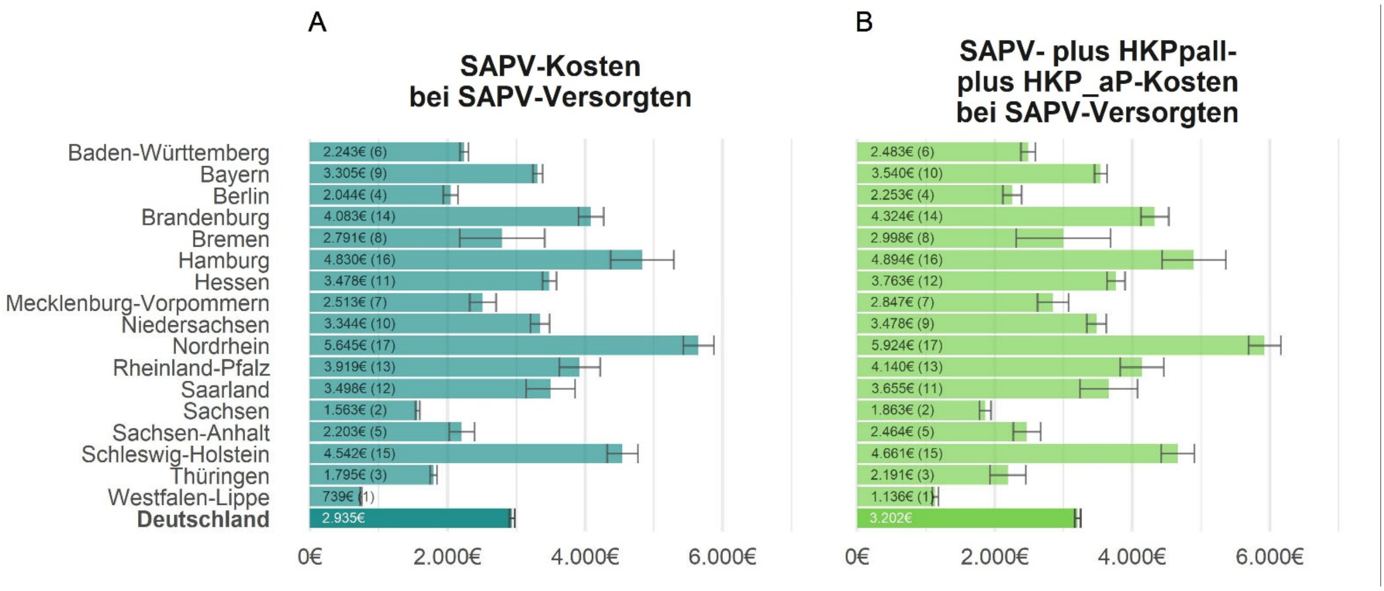

Abbildung B.4-5: A) SAPV-Kosten je Versichertem mit SAPV, B) SAPV-Kosten plus palliative HKP-Kosten[1] je Versichertem mit SAPV (je KV, Mittelwert (Rang), 95%-Konfidenzintervall)

HKP: Häusliche Krankenpflege (SGB V), HKPpall: palliative HKP-Leistungen, HKP_aP: HKP-Leistungen ab erster ambulanter Palliativleistung, KV: Kassenärztliche Vereinigung, SAPV: Spezialisierte ambulante Palliativversorgung

[1]palliative HKP-Kosten = HKPpall + HKP_aP

Quelle: eigene Abbildung

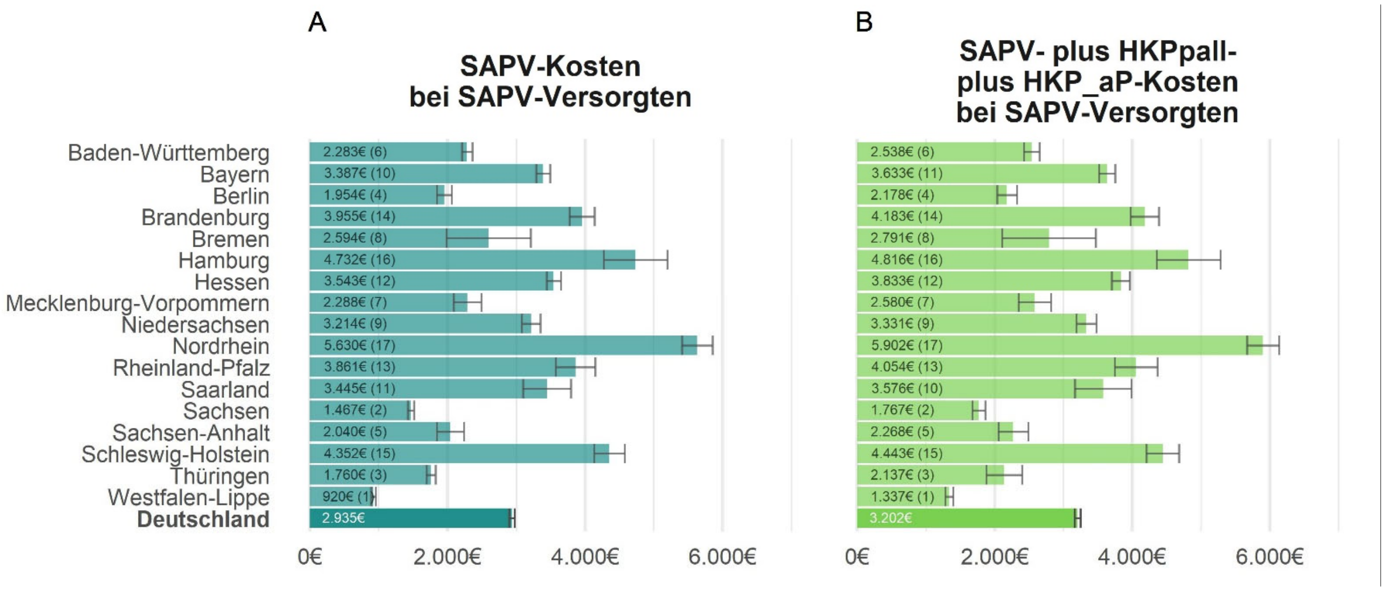

Abbildung B.4-6: A) SAPV-Kosten je Versichertem mit SAPV, B) SAPV-Kosten plus palliative HKP-Kosten[1] je Versichertem mit SAPV (je KV, Mittelwert (Rang), 95%-Konfidenzintervall) - ADJUSTIERT

HKP: Häusliche Krankenpflege (SGB V), HKPpall: palliative HKP-Leistungen, HKP_aP: HKP-Leistungen ab erster ambulanter Palliativleistung, KV: Kassenärztliche Vereinigung, SAPV: Spezialisierte ambulante Palliativversorgung

[1]palliative HKP-Kosten = HKPpall + HKP_aP

Quelle: eigene Abbildung

B.5 Kosten-Effektivitäts-Relationen (KER)

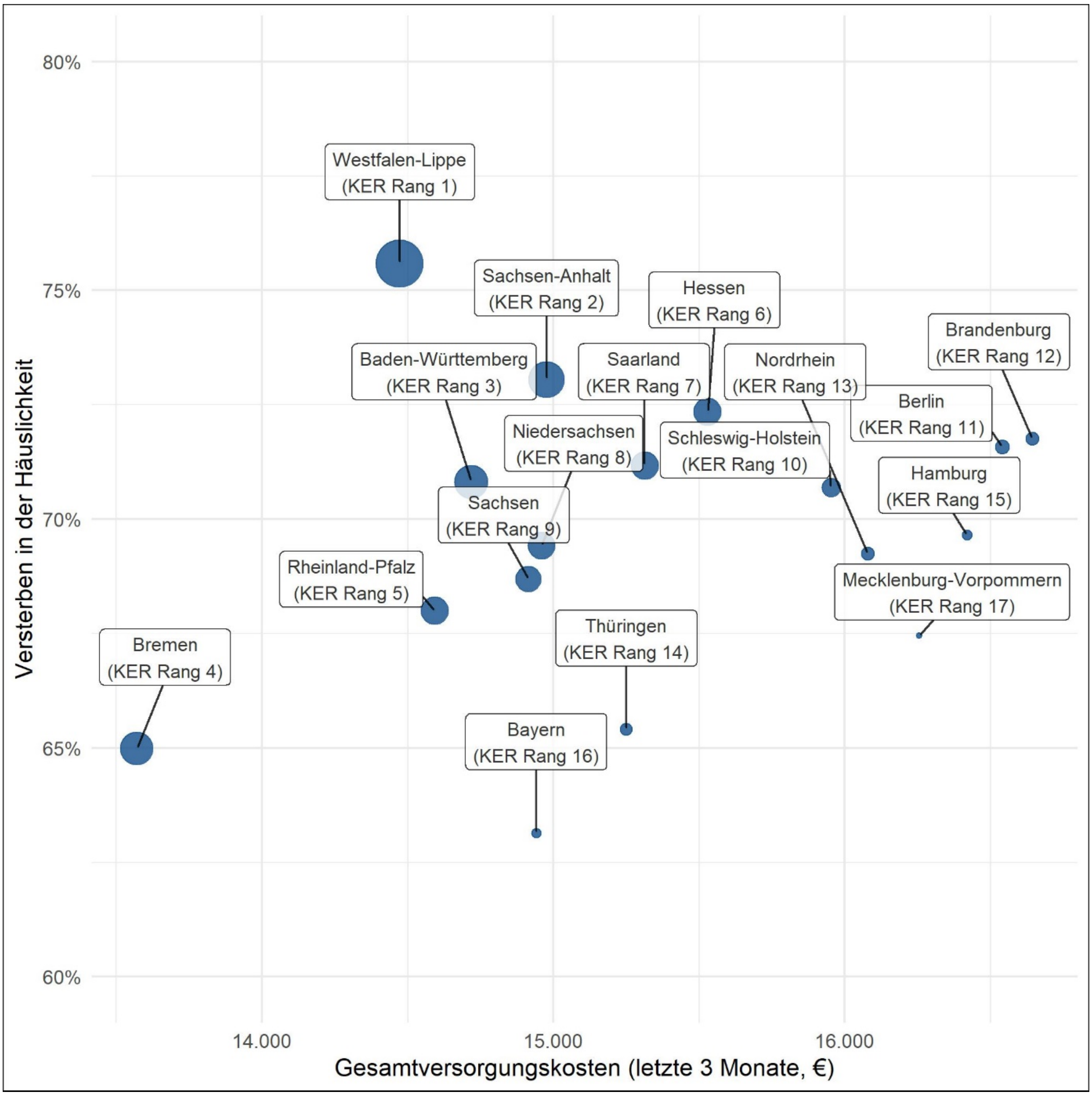

Abbildung B.5-1: Kosten-Effektivitäts-Relation (KER) je KV-Region - ADJUSTIERT

KER als Gesamtversorgungskosten der letzten 3 Lebensmonate je Versichertem zur Rate des Versterbens in der Häuslichkeit für Versicherte mit Palliativversorgung; größere Kreisfläche bedeutet günstigere KER

KV: Kassenärztliche Vereinigung

Quelle: eigene Abbildung

Tabelle B.5-1: Kosten-Effektivitäts-Relation (KER) je KV-Region

	Gesamtkosten (letzte 3 Lebensmonate, €)		Versterben in der Häuslichkeit		KER (€ je 1% Versterben in der Häuslichkeit)		KER (€ je 100% Versterben in der Häuslichkeit)	
**KV**	**Wert**	**Rang**	**Wert**	**Rang**	**Wert**	**Rang**	**Wert**	**Rang**
Baden-Württemberg	14.451	4	71,8%	4	201	3	20.123	3
Bayern	14.444	3	62,4%	17	231	12	23.133	12
Berlin	17.605	16	68,5%	10	257	17	25.686	17
Brandenburg	17.951	17	70,2%	7	256	16	25.564	16
Bremen	13.346	1	66,2%	15	202	4	20.173	4
Hamburg	17.247	15	68,0%	12	254	15	25.370	15
Hessen	15.487	8	73,5%	2	211	5	21.074	5
Mecklenburg-Vorpommern	16.832	14	68,0%	11	247	13	24.746	13
Niedersachsen	14.358	2	71,4%	5	201	2	20.100	2
Nordrhein	15.928	13	70,2%	8	227	11	22.702	11
Rheinland-Pfalz	14.656	6	67,1%	14	218	7	21.838	7
Saarland	15.807	10	69,8%	9	226	10	22.639	10
Sachsen	15.254	7	67,7%	13	225	8	22.539	8
Sachsen-Anhalt	15.595	9	73,2%	3	213	6	21.314	6
Schleswig-Holstein	15.897	12	70,4%	6	226	9	22.571	9
Thüringen	15.817	11	63,8%	16	248	14	24.805	14
Westfalen-Lippe	14.599	5	75,7%	1	193	1	19.280	1
**Deutschland**	**15.317**	**-**	**69,5%**	**-**	**220**	**-**	**22.037**	**-**

KER als Gesamtversorgungskosten der letzten 3 Lebensmonate je Versichertem zur Rate des Versterbens in der Häuslichkeit für Versicherte mit Palliativversorgung
KV: Kassenärztliche Vereinigung

Tabelle B.5-2: Kosten-Effektivitäts-Relation (KER) je KV-Region - ADJUSTIERT

	Gesamtversorgungs-kosten (letzte 3 Monate, €)		Versterben in der Häuslichkeit		KER (€ je 1% Versterben in der Häuslichkeit)		KER (€ je 100% Versterben in der Häuslichkeit)	
**KV**	**Wert**	**Rang**	**Wert**	**Rang**	**Wert**	**Rang**	**Wert**	**Rang**
Baden-Württemberg	14.717	4	70,8%	7	208	3	20.781	3
Bayern	14.941	6	63,1%	17	237	16	23.665	16
Berlin	16.540	16	71,6%	5	231	11	23.110	11
Brandenburg	16.644	17	71,8%	4	232	12	23.195	12
Bremen	13.570	1	65,0%	16	209	4	20.882	4
Hamburg	16.420	15	69,6%	9	236	15	23.575	15
Hessen	15.529	11	72,3%	3	215	6	21.467	6
Mecklenburg-Vorpommern	16.255	14	67,5%	14	241	17	24.098	17
Niedersachsen	14.959	7	69,4%	10	215	8	21.548	8
Nordrhein	16.079	13	69,2%	11	232	13	23.223	13
Rheinland-Pfalz	14.594	3	68,0%	13	215	5	21.464	5
Saarland	15.313	10	71,2%	6	215	7	21.518	7
Sachsen	14.914	5	68,7%	12	217	9	21.714	9
Sachsen-Anhalt	14.976	8	73,0%	2	205	2	20.501	2
Schleswig-Holstein	15.953	12	70,7%	8	226	10	22.568	10

	Gesamtversorgungs-kosten (letzte 3 Monate, €)		Versterben in der Häuslichkeit		KER (€ je 1% Versterben in der Häuslichkeit)		KER (€ je 100% Versterben in der Häuslichkeit)	
**KV**	**Wert**	**Rang**	**Wert**	**Rang**	**Wert**	**Rang**	**Wert**	**Rang**
Thüringen	15.250	9	65,4%	15	233	14	23.319	14
Westfalen-Lippe	14.473	2	75,6%	1	191	1	19.148	1
**Deutschland**	**15.317**	**-**	**69,5%**	**-**	**220**	**-**	**22.037**	**-**

KER als Gesamtversorgungskosten der letzten 3 Lebensmonate je Versichertem zur Rate des Versterbens in der Häuslichkeit für Versicherte mit Palliativversorgung
KV: Kassenärztliche Vereinigung

B.6 Zusammenschau der Variabilität aller Zielindikatoren

Tabelle B.6-1: Variabilität der qualitäts- und kostenbezogenen Outcomeindikatoren zwischen den KV-Regionen

	Zielgröße		Rang 1	Rang 2	Rang 16	Rang 17	Wert Rang 17 / Wert Rang1
Outcomes	Sterbeort KH	rohe Werte	Westfalen-Lippe	Hessen	Thüringen	Bayern	1,55
		Adjustiert	Westfalen-Lippe	Sachsen-Anhalt	Bremen	Bayern	1,51
	Sterbeort KH, nicht Palliativstation	rohe Werte	Saarland	Hessen	Berlin	Mecklenburg-Vorpommern	1,68
		Adjustiert	Saarland	Sachsen	Niedersachsen	Bremen	1,75
	KH-Fall	rohe Werte	Westfalen-Lippe	Berlin	Bremen	Bayern	1,20
		Adjustiert	Berlin	Westfalen-Lippe	Bayern	Bremen	1,27
	KH-Fall, ohne PV	rohe Werte	Sachsen	Westfalen-Lippe	Bayern	Bremen	1,29
		Adjustiert	Thüringen	Sachsen	Bayern	Bremen	1,46
	Rettungsdiensteinsatz	rohe Werte	Westfalen-Lippe	Bayern	Bremen	Thüringen	1,75
		Adjustiert	Bayern	Westfalen-Lippe	Thüringen	Bremen	1,87
	Chemotherapie	rohe Werte	Saarland	Sachsen-Anhalt	Schleswig-Holstein	Hamburg	1,65
		Adjustiert	Saarland	Sachsen-Anhalt	Mecklenburg-Vorpommern	Hamburg	1,61
	ITS-Aufenthalt	rohe Werte	Rheinland-Pfalz	Westfalen-Lippe	Bayern	Berlin	1,81
		Adjustiert	Rheinland-Pfalz	Westfalen-Lippe	Berlin	Mecklenburg-Vorpommern	1,97
	Parenterale Ernährung	rohe Werte	Bayern	Rheinland-Pfalz	Berlin	Brandenburg	4,63
		Adjustiert	Bayern	Rheinland-Pfalz	Berlin	Brandenburg	2,46
	Magensonde (PEG)	rohe Werte	Sachsen	Rheinland-Pfalz	Schleswig-Holstein	Bremen	2,86
		Adjustiert	Rheinland-Pfalz	Sachsen	Schleswig-Holstein	Bremen	2,85
	Aggregierter Outcome	rohe Werte	Westfalen-Lippe	Saarland	Mecklenburg-Vorpommern	Bremen	−0,96
		Adjustiert	Westfalen-Lippe	Saarland	Mecklenburg-Vorpommern	Bremen	−1,36
Kosten	Gesamtversorgungskosten	rohe Werte	Bremen	Niedersachsen	Berlin	Brandenburg	1,35
		Adjustiert	Bremen	Westfalen-Lippe	Berlin	Brandenburg	1,23
	PV-Kosten des letzten Lebensjahrs	rohe Werte	Sachsen	Bremen	Brandenburg	Hamburg	2,38
		Adjustiert	Bremen	Sachsen	Hamburg	Brandenburg	2,31
	SAPV-Kosten bei SAPV-Versorgten	rohe Werte	Westfalen-Lippe	Sachsen	Hamburg	Nordrhein	7,64
		Adjustiert	Westfalen-Lippe	Sachsen	Hamburg	Nordrhein	6,12
	SAPV- plus HKPpall- plus HKP_aP-Kosten bei SAPV-Versorgten	rohe Werte	Westfalen-Lippe	Sachsen	Hamburg	Nordrhein	5,22
		Adjustiert	Westfalen-Lippe	Sachsen	Hamburg	Nordrhein	4,42
KER	KER (basierend auf Gesamtkosten)	rohe Werte	Westfalen-Lippe	Niedersachsen	Brandenburg	Berlin	1,33
		adjustiert	Westfalen-Lippe	Sachsen-Anhalt	Bayern	Mecklenburg-Vorpommern	1,26
Zugespielt	Anteil Nichtversorgter an allen Verstorbenen	rohe Werte	Bayern	Niedersachsen	Bremen	Sachsen-Anhalt	1,30

HKPpall: palliative HKP-Leistungen, HKP_aP: HKP-Leistungen ab erster ambulanter Palliativleistung, ITS: Intensivstation, KER: Kosten-Effektivitäts-Relation, KH: Krankenhaus, KV: Kassenärztliche Vereinigung, PEG: Perkutan-endoskopische Gastrostomie, PV: Palliativversorgung, SAPV: Spezialisierte ambulante Palliativversorgung

C. eDiskussion: Ergänzungen zur Diskussion

C.1 Externe Validität der Outcomes

Die von uns herangezogenen Outcomeindikatoren zur Abbildung der Qualität von PV wurden ähnlich auch in anderen Studien gemessen [5–9]. Die Vergleichbarkeit ist dennoch eingeschränkt, insbesondere, weil andere Einschlusskriterien für die Studienpopulation (Grundgesamtheit) galten. So untersuchte der Bertelsmann Faktencheck [5] für die Jahre 2010-2014 bspw. die Anteile an allen Verstorbenen mit Krebserkrankung, die im letzten Lebensmonat noch eine Chemotherapie erhielten. Diese betrugen dort 7,6% in Bremen und 11,1% in Baden-Württemberg. In Niedersachsen lautete diese Rate 2016/17 auf 10,4% [6]. In unserer Studie schwankt sie zwischen 9,8% im Saarland und 16,1% in Hamburg, allerdings bezogen auf krebserkrankte Verstorbene mit PV im letzten Lebensjahr. Eine Studie zur SAPV in Nordrhein auf der Basis von durch SAPV-Teams dokumentierten Routinedaten [7] ermittelte eine Rate von 85,9% an Versicherten, die im häuslichen Umfeld verstarben. In unserer Studie waren dies in Nordrhein 70,2%, allerdings bei VS, die im letzten Lebensjahr irgendeine Form von Palliativversorgung erhielten. Für die Population der >65-Jährigen variierte der Anteil der in den Jahren 2011-2013 im Krankenhaus Verstorbenen zwischen 41,1% in Baden-Württemberg und 48,8% in Berlin [8]. Im Vergleich liegen in unserer Studienpopulation der VS mit PV Baden-Württemberg unterhalb des bundesdeutschen Mittels und Berlin darüber, die Schwankungsbreite ist aber deutlich größer (vgl. Ergebnisse, Abb. 1). In der Studie von Krause et al. [9] lagen die bundesdurchschnittlichen Raten (2016) für VS, die AAPV oder SAPV erhalten hatten, für ein Versterben im Krankenhaus bei 30,9%, für mindestens einen Krankenhausaufenthalt (ohne solche mit palliativer Versorgung) im letzten Lebensmonat bei 36,5%, Chemotherapie bei Krebserkrankung bei 11,3%, ITS-Aufenthalt bei 4,4%, parenterale Ernährung bei 1,5% und einem Magensondeneinsatz bei 0,6% ggü. entsprechenden vergleichbaren Raten in unserer Studie mit 30,5%, 32,7%, 12,2%, 5,0%, 1,4%, 0,7% bei VS mit PV.

C.2 Externe Validität der Kosten

Direkt vergleichbare Referenzzahlen zu den Gesamtversorgungskosten in den letzten drei Lebensmonaten sind uns nicht bekannt. Sehr wohl einordnen lassen sich die bundesmittleren 15.317€ je VS mit PV (2016-2019) jedoch, wenn man die 21.830€ Gesundheitsausgaben im letzten Lebensjahr heranzieht, die anhand von Daten der AOK Niedersachsen (2017) ermittelt wurden. Die dortigen Gesamtausgaben schließen dieselben Leistungsarten wie unsere Studie ein, jedoch ohne Hospizkosten, aber zzgl. Krankengeld und Rehabilitation [10]. Weitere Referenzzahlen liefert neben eigenen Vorarbeiten [11] eine Studie, die auf DAK-Daten des Jahres 2012 basiert und Gesamtversorgungskosten im letzten Lebensjahr für VS mit AAPV und keiner SAPV von 24.982€ und für VS mit SAPV von 31.744€ ermittelt (wobei hier zusätzlich Pflegeleistungskosten nach SGB XI einbezogen wurden) [12] sowie eine Studie aus Nordrhein, die für Versicherte mit SAPV im letzten Lebensjahr 36.900€ (Jahre 2014-2016) ermittelte [13].

Zu den Palliativversorgungskosten liegen bislang keine deutschen Studien und Auswertungen vor. Dies gilt auch für durchschnittliche Kosten der Leistungen im Rahmen einer Palliativversorgungsform je Patient. Bekannt ist lediglich die öffentliche Berichterstattung zu den jährlichen Ausgaben für SAPV für die GKV bzw. nach Kassenarten [14] sowie die Vergütungssätze für Einzelleistungen, Leistungskomplexe bzw. Fallpauschalen aus den jeweiligen SAPV-Versorgungsverträgen, EBM-Punkte sowie Zusatzentgelte für stationäre Palliativleistungen.

Regional vergleichende Kosten-Effektivitätsstudien zu palliativer Versorgung sind bislang ebenfalls nicht bekannt. International existieren lediglich Vergleiche verschiedener Versorgungsformen

(stationäre Versorgung mit und ohne PV; ambulante versus stationäre palliative Versorgung), bei denen auch inkrementelle Kosten-Effektivitäts-Relationen berechnet werden [15–17].

C.3 Weitere Limitationen der Studie

Weitere Limitationen der angewandten Messung von (Ergebnis-)Qualität

Limitierend ist auch, dass nicht alle gemessenen qualitätsbezogenen Outcomeindikatoren gleichermaßen belastbar sind. So finden sich auf KV-Ebene trotz vier aggregiert betrachteter Jahre bei den Indikatoren ITS-Aufenthalt, parenterale Ernährung und Magensondennutzung innerhalb der letzten 30 Lebenstage nur sehr geringe Raten, hinter denen sich teilweise nur wenige VS verbergen.

Generell sind insbesondere die Ergebnisse für Bremen und das Saarland aufgrund der kleinen Studienpopulationen als eingeschränkt belastbar zu betrachten.

Limitationen bei der Identifikation palliativer Versorgungskosten

Die Erhebung der Palliativversorgungskosten ist dadurch limitiert, dass verschiedene Leistungsarten nicht klar der palliativen Versorgungssituation bzw. den zum Einsatz kommenden palliativen Versorgungsformen zugeordnet werden können. Dies gilt insbesondere für häusliche Krankenpflege, ambulante ärztliche Leistungen, die nicht explizit als palliative Leistungen abgerechnet werden [1] sowie auch für Arzneimittel, Heil- und Hilfsmittel, die zu palliativen Zwecken verordnet werden. Bei der häuslichen Krankenpflege sind wir annäherungsweise so vorgegangen, dass die ab dem Zeitpunkt der ersten abgerechneten Palliativleistung verordnete häusliche Krankenpflege als palliativ veranlasst gewertet wird.

Eine weitere Limitation ist die eingeschränkte Zurechenbarkeit der Vergütung eines Krankenhausfalls (u.a. über DRGs) zu den erbrachten stationären palliativen Versorgungsleistungen. In dieser Studie erfolgt diese Zuordnung ausschließlich anhand der Zusatzentgelte, die nur für einen Teil der Leistungen und teilweise nur für Besondere Einrichtungen gemäß § 17b Absatz. 1 Satz 10 KHG abrechenbar sind [18] und somit zu einer Unterschätzung der GKV-Kosten für stationäre Palliativversorgung führen dürften.

Beide Limitationen sind jedoch nachgeordnet, weil angenommen werden kann, dass sie in allen KV-Regionen gleichermaßen wirken und den KV-Vergleich somit nicht verzerren dürften.

D. Literatur

1. Ditscheid B, Meissner F, Gebel C et al. (2023) Inanspruchnahme von Palliativversorgung am Lebensende in Deutschland: zeitlicher Verlauf (2016-2019) und regionale Variabilität (Utilization of palliative care at the end of life in Germany: temporal trend (2016-2019) and regional variability). Bundesgesundheitsblatt - Gesundheitsforschung - Gesundheitsschutz 66:432–442. https://doi.org/10.1007/s00103-023-03683-7
2. Lumley T (2021) Survey. Analysis of Complex Survey Samples. R Package Version 4.1-1
3. Murtagh FE, Bausewein C, Verne J, Groeneveld EI, Kaloki YE, Higginson IJ (2014) How many people need palliative care? A study developing and comparing methods for population-based estimates. Palliative medicine 28:49–58. https://doi.org/10.1177/0269216313489367
4. Muller CJ, MacLehose RF (2014) Estimating predicted probabilities from logistic regression: different methods correspond to different target populations. Int J Epidemiol 43:962–970. https://doi.org/10.1093/ije/dyu029
5. Radbruch L, Andersohn F, Walker J (2015) Palliativversorgung - Modul 3. Überversorgung kurativ – Unterversorgung palliativ? Analyse ausgewählter Behandlungen am Lebensende. Faktencheck Gesundheit
6. van Baal K, Schrader S, Schneider N et al. (2020) Quality indicators for the evaluation of end-of-life care in Germany - a retrospective cross-sectional analysis of statutory health insurance data. BMC Palliative Care 19:187. https://doi.org/10.1186/s12904-020-00679-x
7. Just J, Schmitz M-T, Grabenhorst U, Joist T, Horn K, Weckbecker K (2022) Specialized outpatient palliative care—clinical course and predictors for living at home until death. Dtsch Arztebl Int:327–332. https://doi.org/10.3238/arztebl.m2022.0172
8. Zich K, Sydow H (2015) Palliativversorgung - Modul 1. Sterbeort Krankenhaus - Regionale Unterschiede und Einflussfaktoren. Faktencheck Gesundheit
9. Krause M, Ditscheid B, Lehmann T et al. (2021) Effectiveness of two types of palliative home care in cancer and non-cancer patients: A retrospective population-based study using claims data. Palliative medicine 35:1158–1169. https://doi.org/10.1177/02692163211013666
10. Stahmeyer JT, Hamp S, Zeidler J, Eberhard S (2021) Gesundheitsausgaben und die Rolle des Alters: Eine detaillierte Analyse der Kosten von Überlebenden und Verstorbenen. Bundesgesundheitsblatt - Gesundheitsforschung - Gesundheitsschutz 64:1307–1314. https://doi.org/10.1007/s00103-021-03385-y
11. Meißner W, Nauck F, Wedding U et al. (2021) SAVOIR - Evaluierung der SAPV-Richtlinie: Outcomes, Interaktionen, Regionale Unterschiede. Ergebnisbericht zum Projekt SAVOIR (01VSF16005), G-BA. Innovationsausschuss
12. Rusche H, Kreimendahl F, Huenges B, Becka D, Rychlik R (2016) Medizinische Versorgung und Kosten im letzten Lebensjahr (Medical care and costs in the last year of life – propensity score matching of AAPV and SAPV insurants). Dtsch med Wochenschr 141:e203-e212. https://doi.org/10.1055/s-0042-114481
13. Meyer I, Schubert I (2020) Inanspruchnahme von SAPV und weiteren palliativen Leistungen, Charakteristika der Leistungsempfänger und Hinweise auf die Zielerreichung von SAPV in der Region Nordrhein. Bericht zur retrospektiven Sekundärdatenanalyse [AP1.1] im Projekt APVEL - Ambulante Palliative Versorgung Evaluieren. In: Krumm N, Rolke R: Evaluation der Wirksamkeit von SAPV in Nordrhein. Ergebnisbericht zum Projekt APVEL (01VSF16007), S 27–134
14. Bundesministerium für Gesundheit KJ 1-Statistik. https://www.bundesgesundheitsministerium.de/themen/krankenversicherung/zahlen-und-fakten-zur-krankenversicherung/finanzergebnisse. Zugegriffen: 3. Mai 2023

15. Hashimoto Y, Hayashi A, Teng L, Igarashi A (2021) Real-World Cost-Effectiveness of Palliative Care for Terminal Cancer Patients in a Japanese General Hospital. J Palliat Med 24:1284–1290. https://doi.org/10.1089/jpm.2020.0649
16. Cartoni C, Breccia M, Giesinger JM et al. (2021) Early Palliative Home Care versus Hospital Care for Patients with Hematologic Malignancies: A Cost-Effectiveness Study. J Palliat Med 24:887–893. https://doi.org/10.1089/jpm.2020.0396
17. Serra-Prat M, Gallo P, Picaza JM (2001) Home palliative care as a cost-saving alternative: evidence from Catalonia. Palliat Med 15:271–278. https://doi.org/10.1191/026921601678320250
18. Fallpauschalen-Katalog. G-DRG-Version 2019
